# Optimized peptide nanofibrils as efficient transduction enhancers for *in vitro* and *ex vivo* gene transfer

**DOI:** 10.3389/fimmu.2023.1270243

**Published:** 2023-11-09

**Authors:** Lena Rauch-Wirth, Alexander Renner, Kübra Kaygisiz, Tatjana Weil, Laura Zimmermann, Armando A. Rodriguez-Alfonso, Desiree Schütz, Sebastian Wiese, Ludger Ständker, Tanja Weil, Dominik Schmiedel, Jan Münch

**Affiliations:** ^1^ Institute of Molecular Virology, Ulm University Medical Center, Ulm, Germany; ^2^ Department for Cell and Gene Therapy Development, Fraunhofer Institute for Cell Therapy and Immunology (IZI), Leipzig, Germany; ^3^ Department Synthesis of Macromolecules, Max Planck Institute for Polymer Research, Mainz, Germany; ^4^ Core Facility Functional Peptidomics, Ulm University Medical Center, Ulm, Germany; ^5^ Core Unit of Mass Spectrometry and Proteomics, Ulm University Medical Center, Ulm, Germany

**Keywords:** transduction enhancer, peptide nanofibrils, gene delivery, CAR-T cells, CAR-NK cells, lentiviral vector, retroviral vector

## Abstract

Chimeric antigen receptor (CAR)-T cell therapy is a groundbreaking immunotherapy for cancer. However, the intricate and costly manufacturing process remains a hurdle. Improving the transduction rate is a potential avenue to cut down costs and boost therapeutic efficiency. Peptide nanofibrils (PNFs) serve as one such class of transduction enhancers. PNFs bind to negatively charged virions, facilitating their active engagement by cellular protrusions, which enhances virion attachment to cells, leading to increased cellular entry and gene transfer rates. While first-generation PNFs had issues with aggregate formation and potential immunogenicity, our study utilized *in silico* screening to identify short, endogenous, and non-immunogenic peptides capable of enhancing transduction. This led to the discovery of an 8-mer peptide, RM-8, which forms PNFs that effectively boost T cell transduction rates by various retroviral vectors. A subsequent structure-activity relationship (SAR) analysis refined RM-8, resulting in the D4 derivative. D4 peptide is stable and assembles into smaller PNFs, avoiding large aggregate formation, and demonstrates superior transduction rates in primary T and NK cells. In essence, D4 PNFs present an economical and straightforward nanotechnological tool, ideal for refining *ex vivo* gene transfer in CAR-T cell production and potentially other advanced therapeutic applications.

## 1 Introduction

Over the past few years, chimeric antigen receptor (CAR)-T cell therapy has gained prominence as an efficient treatment for various hematological malignancies such as B cell lymphoma, B cell acute lymphoblastic leukemia, and multiple myeloma (1–5). CAR-T cells are autologous engineered T cells that target specific tumor antigens leading to an antitumor immune response (6–8). So far, four CAR-T cell products targeting cluster of differentiation 19 (CD19) (axicabtagene ciloleucel (1), tisagenlecleucel (3), lisocabtagene maraleucel (9, 10) and brexucabtagene autoleucel (2)) and two B cell maturation antigen (BCMA)-targeted CAR-T cell products (idecabtagene vicleucel (11) and ciltacabtagene autoleucel (12)) are approved in the US and the EU by the FDA and the European Commission, respectively. All these products are generated by *ex vivo* viral transduction of patients’ T cells using lentiviral or γ-retroviral vectors (13).

Recently, CAR-Natural Killer (NK) cell therapy has gained attention due to its notable advantages compared to CAR-T cell therapy. These advantages include a superior safety profile attributed to a decreased risk of cytokine-release syndrome and neurotoxicity (14), as well as multiple CAR-independent ways to activate cytotoxic activity (15, 16). Furthermore, CAR-NK cell therapy carries a reduced risk for graft-versus-host disease (GvHD), enabling the potential production of a single CAR-NK cell product for multiple patients (17, 18). The viral gene delivery of primary NK cells, which is significantly less efficient compared to primary T cells, poses a significant challenge in using CAR-NK cells for immunotherapy (19).

The manufacturing and administration of CAR-T and CAR-NK cells involve labor-intensive processes and significant costs. One crucial component contributing to these costs is the viral vector required for *ex vivo* cell manufacturing (20). However, by improving the transduction efficiency of target cells, it becomes possible to reduce the quantity of viral vectors needed, leading to potential cost savings (21). Beyond cost reduction, increased transduction efficiency plays a critical role in the success of CAR-T/NK cell therapy, as an adequate number of successfully transduced cells is essential for effective treatment (22). Therefore, focusing on improving transduction rates holds the key to not only potential cost savings but also enhancing the overall success rate of CAR-T and CAR-NK cell therapies.

To enhance transduction efficiency, various transduction enhancers have been developed, including polymers (23), lipids (24), peptides (21, 25), and polypeptides (26). Notably, two transduction enhancers, RetroNectin and Vectofusin-1, have been specifically employed in CAR-T and CAR-NK cell therapies (21, 22, 27, 28). RetroNectin, a recombinant human fibronectin fragment, facilitates lentiviral and γ-retroviral gene transfer by promoting the interaction between virus particles and cells through its heparin-binding domain, as well as its binding to cellular integrin receptors (29). While RetroNectin has demonstrated successful use in generating CAR-T cells (22, 28), its application is time-consuming, requiring pre-coating of cell culture flasks and sometimes centrifugation for improved transduction enhancement (22). On the other hand, Vectofusin-1 offers an alternative transduction enhancer approach. It is a self-assembling cationic 26-mer peptide that forms α-helical nanofibrils (30). Vectofusin-1 has shown efficacy in enhancing γ-retroviral and lentiviral transduction rates and is implemented in an automated and closed system, such as the CliniMACS Prodigy™, for CAR-T cell production (21).

Another promising transduction enhancer is Protransduzin, previously described as enhancing factor C (EF-C), a 12-mer peptide derived from Human immunodeficiency virus 1 (HIV-1) glycoprotein gp120, that forms β-sheet peptide nanofibrils (PNF) (25). These PNFs possess a positive surface charge, enabling them to effectively capture negatively charged viral particles. This interaction enhances the attachment of the viral particles to target cells, leading to increased transduction rates (25). Studies have further revealed that Protransduzin’s transduction enhancing activity is actively mediated by cellular protrusions. This mechanism explains its superior performance compared to other additives (31). Protransduzin offers advantages over other transduction enhancers, such as Vectofusin-1 and RetroNectin, due to its shorter length and independence from additional steps like centrifugation or coating. This makes it a more cost-effective and user-friendly alternative. However, the chemical instability of Protransduzin, caused by the cyclization of its N-terminal glutamine to pyroglutamate, presents a challenge, which leads to significant aggregate formation in cell culture (32).

In this study we screened for novel short endogenous and non-immunogenic peptides that assemble into PNFs that efficiently enhance viral transduction rates but do not form large aggregates in cell culture, using an *in silico* screening approach. We discovered an 8-mer peptide, termed RM-8, derived from human interleukin-18, that forms chemically stable β-sheet PNFs, which effectively boost γ-retroviral and lentiviral gene transfer. By applying a structure-activity relationship (SAR) study RM-8 was optimized regarding aggregate formation to its derivative D4, which shows comparable or better transduction rates in primary T and NK cells than RM-8, Vectofusin-1, and EF-C. Thus, D4 PNFs represent a novel, easy-to-apply nanotechnological tool to increase viral transduction rates in the laboratory, and to optimize the production of CAR-T and CAR-NK cells.

## 2 Materials and methods

### 2.1 Cell culture

TZM-bl (AIDS reagent and reference programme, Saic Frederick, USA) and HEK293T cells (ATCC, CRL-3216) were cultured in Dulbecco´s Modified Eagle Medium (DMEM, Gibco, Catalog number (Cat): 11965092) supplemented with 10% inactivated fetal calf serum (FCS, Gibco, Cat: 10437028), 2 mM L-glutamine (PAN-Biotech, Cat: P04-80050), 100 U/ml penicillin and 100 µg/ml streptomycin (PAN-Biotech, P06-07050). Jurkat cells (ATCC, TIB-152) and THP1-Dual cells (InvivoGen, Cat: thpd-nfis) were cultured in Roswell Park Memorial Institute 1640 Medium (RPMI, Gibco, Cat: 11875093) supplemented with 10% FCS, 2 mM L-glutamine, 100 U/ml penicillin and 100 µg/ml streptomycin. T cells were cultured in RPMI medium supplemented with 10% FCS, 2 mM L-glutamine, 100 U/ml penicillin, and 100 µg/ml streptomycin and different interleukins as described below.

### 2.2 Cell viability

To determine the effect of peptides on cellular metabolic activity, MTT (Thiazolyl blue tetrazolium bromide) Cell Growth Assay (Merck, Cat: CT02) and CellTiter-Glo Luminescent Cell Viability Assay (Promega, Cat: G7571) were performed. 10,000 TZM-bl cells or 50,000 Jurkat cells were seeded into 96-well plates and treated with indicated concentrations of peptides. For TZM-bl cells, the supernatants were removed after three days and 100 µl MTT (0.5 mg/ml) was added and incubated for 2.5 hours. For Jurkat cells, supernatants were removed after two days, and cells were incubated with 40 μl MTT (1 mg/ml) for 5 hours. Then the MTT solution was removed, and 100 µl 1:1 DMSO-Ethanol was added. The absorption was measured at 590 nm with baseline-corrected at 650 nm using VersaMax Microplate Reader (Molecular Devices). The CellTiter-Glo assay was performed according to the manufacturing protocol. 100 μl CellTiter-Glo Reagent 1:1 diluted in PBS (Gibco, 14200075) was added to pelleted Jurkat cells for 10 min followed by transferring 50 µl to white microplate, and luminescence was recorded by Orion microplate luminometer (Berthold).

### 2.3 *In-silico* screening for endogenous peptide nanofibrils (PNFs)

The Human proteins serum albumin (HSA, P02768), glyceraldehyde-3-phosphate dehydrogenase (GAPDH, P04406), signal transducing adapter molecule 2 (Stam2, O75886), and interleukin-18 (IL-18, Q14116) were analyzed by the amyloid prediction tools ZipperDB (33), Tango (34–36) and PASTA 2.0 (37). The amino acid sequence of the above proteins derived from UniProt was used. With the help of the ZipperDB the Rosetta energy was determined to predict fibrillation propensity, and hexapeptides showing a Rosetta energy below -23 kcal/mol were chosen based on Goldschmidt et al., 2010 (33). Additionally, the statistical mechanics algorithm TANGO was used. Fernandez-Escamilla et al., 2004 demonstrated that a TANGO score (percentage β-aggregation per residue) above 5% in a window of at least five residues is a good predictor for aggregation (35), which was also used for the analysis of HSA, GAPDH, Stam2, and IL-18. The following conditions were used for TANGO analysis: pH = 7, temperature = 25°C, ionic strength = 0.02 M, and concentration = 1 M. Using the server PASTA 2.0 for the analysis, an energy cut-off -5 Pasta Energy Unit (PEU) based on Walsh et al., 2014 (37), and a top pairing energy of 20 was selected for the analysis of the above-mentioned proteins. 1 PEU corresponds to 1.192 kcal/mol. The molecular weight and theoretical pI of the endogenous peptides were determined using ProtParam, a tool for the computation of various physical and chemical parameters (38).

### 2.4 Preparation of peptide nanofibrils

Peptides were synthesized by KE Biochem Co. (Shanghai City, China), Core Facility Functional Peptidomics (Ulm University Medical Centre, Ulm, Germany) and GaloreTx Pharmaceuticals (Udupi, India). Peptides were dissolved in DMSO (Merck, Cat: 67-68-5) to 10 mg/ml and stored at 4°C. For each experiment, the peptides were freshly dissolved in PBS to 2 mg/ml and incubated for 10 min at room temperature to generate peptide nanofibrils (PNFs). Unless otherwise described, this concentration was used for the experiments. Vectofusin-1 (Miltenyi Biotec, Cat: 130-111-163) was solved in sterile water to 1 mg/ml and stored at -80°C according to manufacturing protocol.

### 2.5 Thioflavin T (ThT) assay

To monitor the presence of PNFs, 25 μl PNFs solution was mixed with 24 μl PBS and 1 μl 2.5 mM ThT (Sigma, Cat: T3516) and incubated for 10 min at dark before fluorescence was measured at an excitation wavelength of 450 nm and an emission endpoint of 482 nm using a Synergy H1 hybrid multi-mode reader (Biotek).

### 2.6 Zeta potential

To determine the surface charge of fibrils, the zeta potential was measured. PNFs solutions were diluted in 1 ml 1 mM KCl (Merck, Cat: 104936) so that a number of traced particles of 100-400 was obtained. The zeta potential derived from the electrophoretic mobility of the PNFs was measured using the ZetaView TWIN (Particle Metrix) and the corresponding Software ZetaView (Zetaview Nanoparticle Tracking Analyzer RRID: SCR_016647).

### 2.7 Nanoparticle tracking analysis (NTA)

NTA to determine the size distribution of PNFs was performed using ZetaView TWIN. 2 mg/ml PNFs and 1 mg/ml Vectofusin-1 solutions were diluted in particle-free PBS, and videos of the light-refracting particles were recorded with the following settings: 25°C fixed temperature, 11 positions, 1 cycle, sensitivity 70, shutter 100, 15 fps, 2 s videos/position, 3 measurements. The chamber was rinsed between the samples with particle-free PBS.

### 2.8 Attenuated total reflection Fourier transform infrared (ATR-FTIR) spectroscopy

To analyze the secondary structure PNFs solutions were freshly prepared by diluting the DMSO stock (c = 10 mg/ml) tenfold using PBS (c = 1 mg/ml). 200 µl of samples were lyophilized, and all spectra were recorded on a Bruker Tensor 27 spectrometer with a diamond crystal as ATR element (PIKE MiracleTM, spectral resolution 2 cm^-1^) according to a previous report (39).

### 2.9 Conversion rate (CR)

The CR determines the ratio of peptide monomers that convert into aggregated structures. To measure the CR, 1 mg/ml of PNFs were incubated in PBS for one day and the assay was performed according to a previous report (40).

### 2.10 Transmission electron microscopy (TEM)

5 μl PNFs solution (2 mg/ml) was incubated on a glow-discharged carbon copper grid for 5 min. The liquid on the grid was removed by Whatman™ filter paper (Merck) followed by three washing steps using 10 μl water each and then stained three times with 10 μl 0.5% (w/v) uranyl acetate (Merck). Remaining uranyl acetate was removed, and the grid was dried. Samples were imaged with the JEM-1400 120kV transmission electron microscope (Jeol).

### 2.11 Microscopic analysis of PNFs

To visualize aggregates of PNFs, microscopic images were taken of 2 mg/ml PNFs using Cytation 5 and 4x phase contrast (BioTek).

### 2.12 High-performance liquid chromatography (HPLC) analysis

RM-8 and D4 were prepared at 2 mg/ml in PBS and were incubated at room temperature (RT) or 37°C for 10 min, 1 hour, 24 hours, and 10 days. Aliquots (25 µL) of both samples were separated after 10 minutes, 1 hour, 24 hours, and 10 days and centrifuged at 13,000 x g for one minute. Supernatants were diluted with 100 µL 10% acetic acid, whereas the pellets were dissolved in 125 µL 6 M guanidinium chloride. All samples were analyzed in an Agilent 1100 Series HPLC system, using a reversed-phase BioBasicTM 18-HPLC column (Thermo Scientific) of dimensions 2.1 x 100 mm and particle size of 5 µm. Gradient elution was used for elution as follows (RM-8): 0 min/5%B, 1 min/20%B, and 15 min/40%B; and for D4: 0 min/5%B, 1 min/15%B, and 15 min/35/%B), being A, 0. 1% TFA in water, and B, 0.1% TFA in acetonitrile. The flow rate was 0.5 mL/min, and the detection was monitored online by UV absorption at 214 nm. ChemStation (version B.04.03, Agilent) was used for data acquisition and the HPLC system’s control.

### 2.13 Matrix assisted laser desorption ionization-time of flight mass spectrometry (MALDI-TOF MS) analysis

Mass spectra were obtained with an Axima Confidence MALDI-TOF mass spectrometer (Shimadzu). Measurements were performed in positive ion reflectron mode. The mass range was 100–10000 Da. For each sample, 20 shots were accumulated per profile (100 profiles per spectra) using a laser power of 42% and a laser frequency of 50 Hz. TOFMix MALDI kit (Shimadzu) was used for external mass calibration. Shimadzu Biotech Launchpad software (version 2.9.8.1, Kratos Analytical, UK) was used for spectra visualization and equipment control.

### 2.14 Virus stocks

For virus stock generation, 800,000 HEK293T cells were initially seeded, followed by transfection using different plasmids and TransIT-LT1 Transfection Reagent (Mirus Bio, Cat: Mir 2305) according to the manufacturer’s protocol on the next day. After two days, virus stocks were harvested, centrifuged (3 min at 1300 rpm) and supernatants were stored at -80°C. Infectious HIV-1 stocks were produced by transfection of HEK293T cells using pBRNL4.39-92TH14 (5 µg), a plasmid containing a CCR5 tropic molecular HIV-1 clone (41). For GALV γ-retroviral vector (GALV-RV) production, HEK293T cells were cotransfected with the following plasmids: GFP expressing Murine leukemia virus (MLV) vector E200 pcmE26-gfp (1.15 µg), E848 pCsGPpA-ed (0.95 µg) and glycoprotein derived from Gibbon ape Leukemia Virus (GALV, 0.4 µg) (39). GALV-RV was titrated on Jurkat cells and transduction efficiency was determined by flow cytometry after two days. The GALV-RV titer was determined as transducing units per volume (TU/ml). GFP-expressing VSV-G pseudotyped lentiviral vector (VSV-G-LV) was generated by cotransfection of HEK293T cells with the plasmids pRSV-rev (0.25 µg, Addgene plasmid # 12253, a gift from Didier Trono) (42), pRRL.cPPT.SF-eGFP.pre (1 µg) (25), pMDLg/pRRE (3 µg, Addgene plasmid # 12251, a gift from Didier Trono) (42) and Vesicular Stomatitis Virus glycoprotein (VSV-G, 0.5 µg). Luciferase expressing VSV-G-LV was generated by cotransfection of HEK293T cells with the plasmids pSEW-luc2 (2 µg) and pCMV-dR8.91 (2 µg) and VSV-G(1 µg) (39). Luciferase-expressing RD114/TR pseudotyped lentiviral vector (RD114/TR-LV) was generated by cotransfection of HEK293T cells with an env deleted pBRHIV-1 NL4_3 derivative (2.5 µg) encoding luciferase instead of nef and phCMV-RD114/TR (0.31 µg) encoding chimeric envelope glycoproteins derived from Feline Leukemia Virus (RD114) with the cytoplasmic tail derived from the MLV glycoprotein (25). For RD114/TR γ-retroviral vector (RD114/TR-RV) production, HEK293T cells were cotransfected with the following plasmids: GFP expressing Murine leukemia virus (MLV) vector E200 pcmE26-gfp (1.15 µg), E848 pCsGPpA-ed (0.95 µg) and RD114/TR glycoprotein (0.4 µg). RD114/TR-RV was titrated on HEK293T cells and transduction efficiency was determined by flow cytometry after two days. GFP-encoding lentiviral vectors (LV) were generated by transfection of HEK293T cells (150,000 cells were seeded 1 day prior transfection) using following plasmids: 0.667 µg of pMDLg/pRRE-gagpol (Addgene plasmid # 12251, a gift from Didier Trono), 0.167 µg pRSV-Rev (Addgene plasmid # 12253, a gift from Didier Trono) (42), 1 µg of pCDH-CMV-MCS-EF1-GreenPuro (System Biosciences, Cat: CD513B-1) and an envelope plasmid (0.167 µg) for pseudotyping. RD114-TR (pLTR-RD114A, Addgene plasmid # 17576, a gift from Jakob Reiser) (43) or baboon envelope (BaEV, pTwist-BaEVRless) (44) were used as envelope proteins. Two days after transfection, the cell culture supernatant was collected and filtered using a 0.45 µm syringe filter. LV containing supernatants were stored at -80°C.

### 2.15 Effect of PNFs on HIV-1 infection

To determine the effect of PNFs on HIV-1 infection 10,000 TZM-bl cells, which contain the reporter gene β-galactosidase under the control of the HIV-1 long terminal repeat (LTR) promoter, were seeded in a 96-well plate in 180 µl supplemented DMEM one day prior to infection. The cells were inoculated with 20 µl PNFs solution 1:1 (v/v) mixed with virus (2500 dilution on cells) after an incubation time of 10 min to allow the binding of virus particles to PNFs. Three days post HIV-1 infection, the cell culture medium was removed, and 42 µl of 1:8 diluted Gal-screen substrate (Thermo Fisher, Cat: T1027) in PBS was added. After incubation for 30-40 min, 36 µl of lysates were transferred to white microtiter plates, and β-galactosidase activity was recorded as relative light units (RLU) by Orion II Microplate Luminometer (Berthold).

### 2.16 Effect of PNFs on lentiviral transduction

To detect transduction rates of luciferase encoding lentiviral vectors (VSV-G-LVs, RD114/TR-LVs), 10,000 HEK293 cells were seeded in a 96-well plate in 180 µl supplemented DMEM one day prior to infection. The cells were inoculated with 20 µl PNFs solution 1:1 (v/v) mixed with virus (1:640 dilution on cells for VSV-G-LV and 1:80 dilution on cells for RD114/TR-LV) after an incubation time of 10 min. Two days after VSV-G-LV infection and three days after RD114/TR-LV infection the supernatant of cells was removed and cells were lysed in 40 µl 1-fold diluted Luciferase Cell Culture Lysis Reagent (Promega). After 10 min, cells were resuspended and 30 µl of the lysates were transferred into white microtiter plates. Thereafter, 50 µl luciferase substrate reconstituted in luciferase buffer (Luciferase Assay system, Promega) was added, and luminescence was measured as RLU using Orion II Microplate Luminometer (Berthold).

### 2.17 Effect of PNFs on γ-retroviral transduction

For the determination of infectivity enhancement of GALV-RV, 50,000 Jurkat cells were seeded in 180 µl supplemented RPMI. The cells were inoculated with 20 µl PNFs solution 1:1 (v/v) mixed with GALV-RV after an incubation time of 10 min. Two days post infection, GFP-expression was analyzed by flow cytometry using CytoFlex (Beckmann Coulter) and the evaluation was carried out with FlowJo™ (Version 10.8.1) according to the gating strategy shown in **Figure S1**. For analyzing the transduction enhancement of RD114/TR-RV, 10,000 HEK293T cells were seeded in a 96-well plate in 100 µl supplemented DMEM one day prior to infection. The cells were inoculated with 100 µl PNFs solution 1:1 (v/v) mixed with virus after an incubation time of 10 min. Transduction efficiency was determined three days later by flow cytometry.

### 2.18 γ-retroviral and lentiviral transduction of human T cells

Human CD4+ and CD8+ T cells were isolated from Buffy coats of healthy donors using RosetteSep™ Human CD4+ Enrichment Cocktail (STEMCELL Technologies, Cat: 15062) and CD8+ Enrichment Cocktail (STEMCELL Technologies, Cat: 15063) according to manufacturer’s protocol. Isolated T cells were activated using Dynabeads™ Human T-Activator CD3/CD28 (Gibco, Cat: 11131D) according to manufacturer’s protocol in supplemented RPMI in presence of 10 ng/ml IL-2 (Miltenyi Biotec, Cat: 130-097-748) for three days. The purity of isolated T cells was determined by flow cytometry using CD4, CD8, and CD11c antibodies and their corresponding isotype control (**Table S1**). After three days, the magnetic Dynabeads were removed, and T cells were cultured in supplemented RPMI in presence of 450 IU/ml IL-7 (Miltenyi Biotec, Cat: 130-095-362) and 50 IU/ml IL-15 (Miltenyi Biotec, Cat: 130-095-764). After one day 5×10^5^ T cells were transduced with GALV-RV (1:4 dilution on cells) or VSV-G-LV (1:40 dilution on cells) in absence and presence of RM-8 (30 or 50 µg/ml), D4 (30 µg/ml), EF-C (30 or 50 µg/ml), 10 µg/ml Vectofusin-1 (Miltenyi Biotec, Cat: 130-111-163), or 20 µg/ml RetroNectin (Takara Bio, Cat: T100B). The 24-well plate was coated with RetroNectin and incubated overnight at 4°C. Wells were blocked with PBS containing 2% BSA (Gibco, Cat: 15260037) for 30 min and washed twice with PBS. Virus was centrifuged onto coated wells at 2,000 g at 32°C for 2 h. Viral supernatant was removed and T cells were added and subsequently spin-infected (300 g for 10 min). One day after transduction, the medium was exchanged. T cells were cultured with a cell density of 1×10^6^ cells/ml for seven days. T cells were analyzed for CD3 expression using an antibody and its corresponding isotype controls shown in **Table S1**. Furthermore, viability using LIVE/DEAD™ Fixable Aqua Dead Cell Stain Kit (ThermoFisher Scientific, Cat: L34957) and Trypan blue stain (Invitrogen, Cat: T10282) according to the manufacturer’s protocol and transduction efficiency (% GFP+ cells) were determined. Flow cytometry analysis were performed using CytoFlex (Beckmann Coulter) and evaluation was carried out with FlowJo™ (Version 10.8.1). The gating strategy shown in **Figure S2** was applied.

### 2.19 Lentiviral transduction of human NK cells

NK cells were isolated from buffy coats of healthy donors by standard density-gradient centrifugation, using Ficoll-Paque (VWR, Cat: 17-1440-03) using the RosetteSep™ Human NK Cell Enrichment Cocktail (STEMCELL Technologies, Cat: 15065). NK cells were cultured at 1×10^6^ cells/ml in NK MACS medium (Miltenyi Biotec, Cat: 130-114-429) with 5% human AB serum (Sigma Aldrich, Cat: H4522-100ML), 500 U/ml IL-2 (Peprotech, Cat: 200-02) and 140 U/ml IL-15 (Peprotech, Cat: 200-15). After seven days of culturing, NK cells were transduced with lentiviral particles. 1.25×10^5^ NK cells were seeded per well in a 48-well plate. 250 µl of lentiviral supernatant (undiluted RD114/TR-LV stock, 1:3 diluted BaEV-LV stock in HEK293T culture medium) was mixed with transduction enhancers to final concentrations of 10 µg/ml for Vectofusin-1 and either 10, 20 or 30 µg/ml for RM-8 and D4 peptides and then incubated at room temperature for 10 min prior to adding to the NK cells. After incubation at 37°C for 1 h, 250 µl of fresh complete NK MACS medium was added to the transduced cells. For spinfection, cells were centrifuged (400 g, 1 h at 37°C) after virus and transduction enhancer were added. Transduction efficiency (GFP+ cells) was assessed four days after transduction using flow cytometry (MACSQuant10, Miltenyi Biotec). Propidium Iodide (PI) (Miltenyi Biotec, Cat: 130-093-233) staining was used to determine the viability of NK cells.

### 2.20 PNFs stimulation of THP1-Dual cells

To analyze if PNFs stimulate an immune response, different PNFs concentrations (5, 10, 30 and 50 µg/ml) and 0.1 µg/ml lipopolysaccharide (LPS, InvivoGen, Cat: tlrl-eklps) as a positive control were incubated with 70,000 THP1-Dual cells, a monocyte cell line that allows the simultaneous analysis of the NF-κB and interferon regulatory factor (IRF) pathway, which are major effectors of the innate immune response (45). After 24 h, cellular supernatants were analyzed for the activation of the NF-κB pathway by measuring the activity of secreted alkaline phosphatase (SEAP). Therefore, 20 µl supernatant was mixed with 120 µl Alkaline Phosphatase Blue Microwell Substrate (Sigma Aldrich, Cat: AB0100-1KT). After 10 min, 30 µl Alkaline Phosphatase Stop Solution (Sigma Aldrich, Cat: A5852) was added and adsorption at 620 nm was measured using VersaMax Microplate Reader (Molecular Devices). For analysis of IRF pathway, secreted Gaussia Luciferase was measured. 25 µl supernatant was transferred to a white 96 well microtiter plate and Gaussia substrate (82.6 µg/ml Colenterazine, PJK, Cat: 102172) was automatically added according to manufacturer’s protocol and Luminescence was measured by Orion II Microplate Luminometer (Berthold).

### 2.21 PNFs stimulation of CD4+ T cells

For the investigation of T cell immune response after PNFs stimulation, 50,000 CD4+ T cells were incubated with different PNFs concentrations (5, 10, 30 and 50 µg/ml) and 0.1 µg/ml LPS as a positive control. After 24 h, supernatants were analyzed by flow cytometry, using the LEGENDplex Human Inflammation Panel 1 (Biolegend, Cat: 740809), a bead-based multiplex assay to quantify human inflammatory cytokines/chemokines, according to manufacturing protocol. Mean fluorescence of the samples were normalized to the untreated control.

### 2.22 Statistics

Statistical analysis including one-way ANOVA with Dunnett’s multiple comparison test were performed using GraphPad Prism version 9.5.1.

### 2.23 Ethics statement

NK cells from healthy donors were isolated from buffy coats acquired from the blood bank of the University Hospital Leipzig; ethic vote number 327/22-ek. T cells from healthy donors were isolated from buffy coats acquired from the Blutspendezentrale Ulm; ethic vote number 151/22 FSt/bal.

## 3 Results

### 3.1 *In silico* screening for peptide nanofibrils as transduction enhancer

We previously identified the 12-mer peptide EF-C that self-assembles into peptide nanofibrils (PNFs) and efficiently enhances γ-retroviral gene transfer in various *in vitro* settings (25). The application of EF-C for CAR-T cell generation is however limited due to its time-dependent chemical instability and the formation of large aggregates under cellular conditions (32). Additionally, its sequence is derived from HIV-1 glycoprotein gp120 (25), which may potentially induce immunogenicity. Therefore, our goal was to identify novel endogenous and non-immunogenic peptides that assemble into PNFs and efficiently enhance viral transduction without forming larger aggregates.

**Figure 1:**
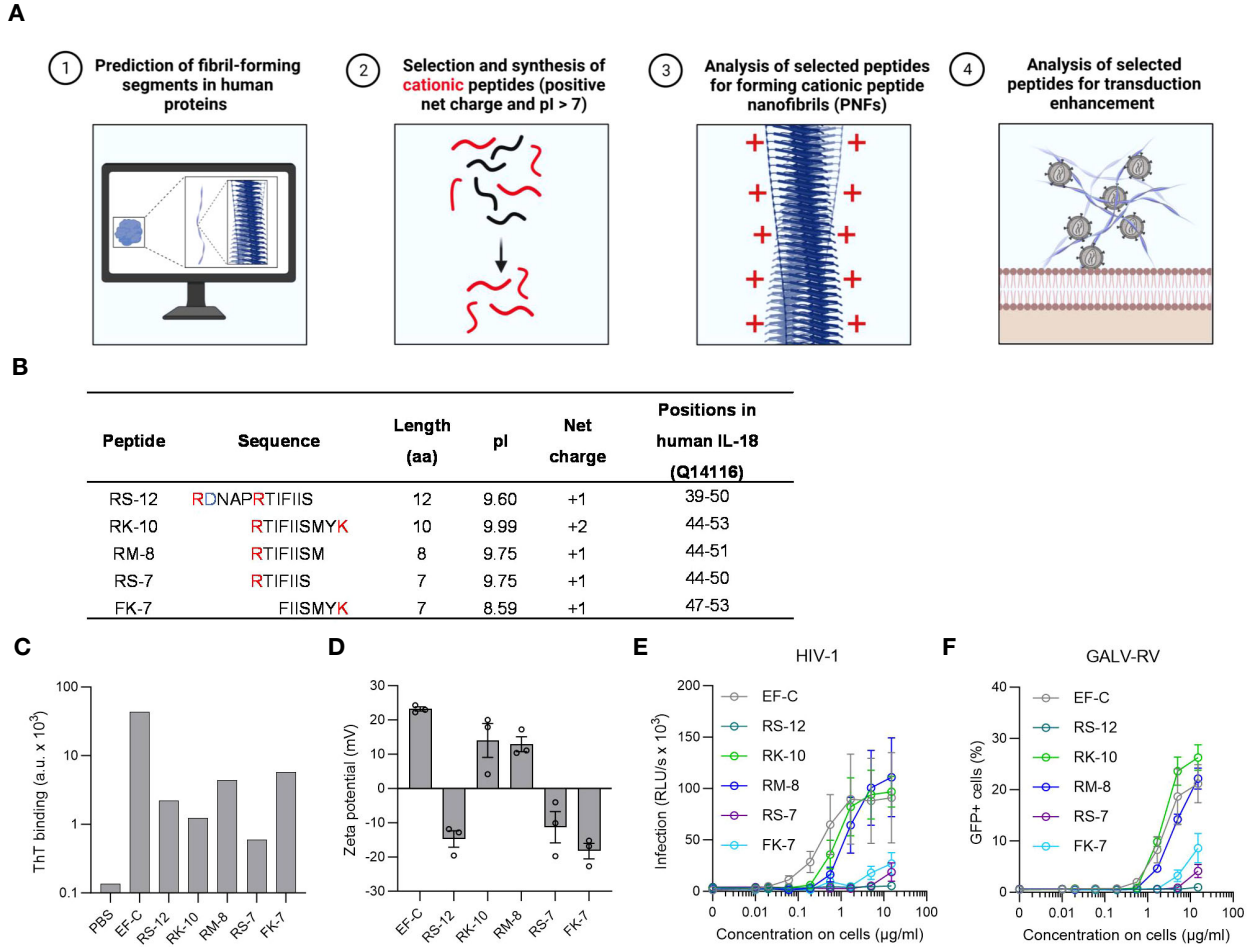
*In silico* identification and characterization of IL-18 derived PNF as potential enhancers for viral transduction. **(A)** *In silico* screening for endogenous PNFs as transduction enhancers using different amyloid aggregation algorithms (Tango, PASTA 2.0, and ZipperDB). Created with BioRender.com. **(B)** Physical and chemical parameters of selected IL-18 derived peptides with positive net charge based on *in silico* screening results. The positively charged amino acids are marked in red, and the negatively charged residues in blue. The positions of the peptides correspond to human IL-18 (UniProtKB: Q14116) without the propeptide. **(C)** ThT fluorescence intensities of tested peptides. Shown is one measurement from one experiment. **(D)** Zeta potential, an indicator of the surface charge, of IL-18 derived PNFs. The results shown are average values (± SEM) of duplicates measurement from three independent experiments. **(E)** RM-8 and RK-10 PNFs increase HIV-1 infection of TZM-bl cells. HIV-1 was treated with PNFs and mixtures were used to infect TZM-bl cells. Tat-inducible β-galactosidase activity was measured three days later. The results shown are average values (± SEM) of triplicate measurements from three independent experiments. **(F)** RM-8 and RK-10 PNFs enhance GALV-RV infection of Jurkat cells. GFP-expressing GALV-RV was treated with PNFs and mixtures were used to infect Jurkat cells. GFP positive (GFP+) cells were determined two days later by flow cytometry. The results shown are average values (± SD) of three independent experiments. GALV, glycoprotein of gibbon ape leukemia virus; HIV-1, Human immunodeficiency virus 1; IL-18, interleukin-18; PNFs, peptide naofibrils; RLU/s, relative light units per second; RV, γ-retroviral vector; ThT; Thioflavin T; SEM, standard error of the mean.

For this, we analyzed arbitrary human proteins (serum albumin (HSA), glyceraldehyde-3-phosphate dehydrogenase (GAPDH), signal transducing adapter molecule 2 (Stam2) and interleukin-18 (IL-18)) by an *in silico* screening approach using a combination of amyloid prediction algorithms (**Figure 1A**). As selection criteria, proteins were chosen according to their expression (high: HSA and GAPDH, low: Stam2 and IL-18) and localization (extracellular: HSA, IL-18, intracellular: GAPDH, Stam2). The algorithms Tango, PASTA 2.0, and ZipperDB collectively offer predictive capabilities for identifying various aspects of protein aggregation and have been evaluated by us in a previous report to perform well for the prediction of short self-assembling peptides (46). TANGO predicts aggregation areas in unfolded polypeptide chains (34–36), PASTA 2.0 predicts amyloid fibril regions in proteins (37), and ZipperDB predicts fibril-forming segments capable of forming the steric zipper, the backbone of an amyloid fibril (33). By *in-silico* screening, a fibril-forming segment (position 39-53) was determined for IL-18 by all three algorithms (**Figure S3**). For the other proteins only a hexapeptide in HSA was predicted to self-assemble which could not be experimentally confirmed.

Based on the predicted region in IL-18 five different cationic peptides named RS-12, RK-10, RM-8, RS-7, and FK-7 were chemically synthesized and further analyzed (**Figure 1B**). PNFs formation of the five peptides was achieved by dilution of their DMSO stock (10 mg/ml) in PBS (2 mg/ml) and incubation for 10 min at room temperature (RT). To test the peptides’ ability to form PNFs, they were incubated with the amyloid dye Thioflavin T (ThT), showing a clear ThT fluorescence for all peptides (**Figure 1C**). Since we previously showed that the positive charge of PNFs is essential for their transduction enhancing property (25, 39, 47), the zeta potential (ZP), an indicator for the surface charge, was measured. ZP measurements revealed a positive ZP for RK-10 and RM-8 PNFs (**Figure 1D**). In fact, both PNFs efficiently enhanced HIV-1 infection of TZM-bl cells (**Figure 1E**) while not being cytotoxic (**Figure S4A**). Moreover, RM-8 PNF also increased transduction rates of T cells with a γ-retroviral vector pseudotyped with the glycoprotein of gibbon ape leukemia virus glycoprotein (GALV-RV), within a comparable range to EF-C PNFs, while exhibiting no cytotoxic effects (**Figures 1F**, **S4**).

### 3.2 RM-8 PNFs efficiently enhance γ-retroviral and lentiviral transduction

**Figure 2:**
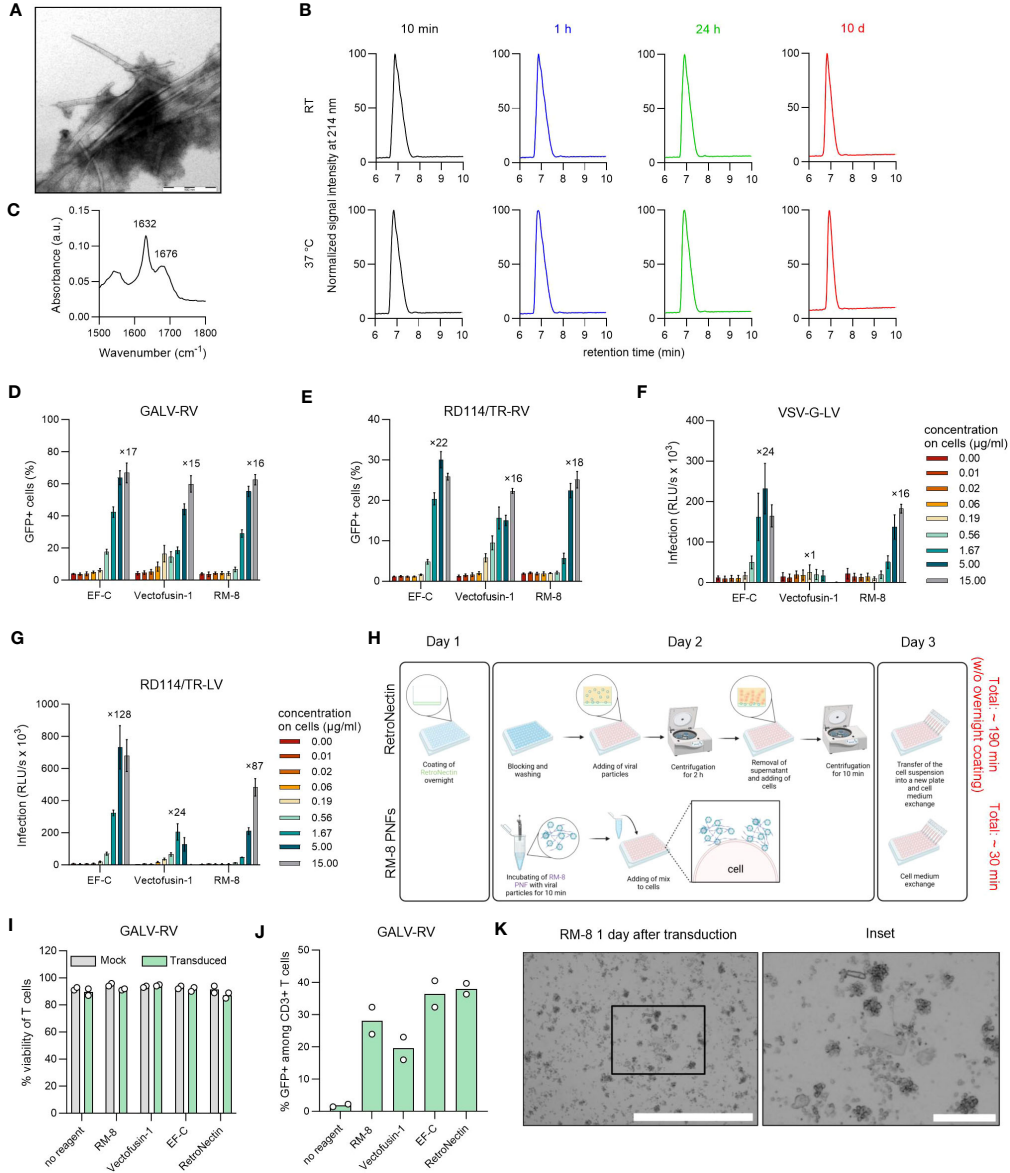
Biophysical and functional characterization of RM-8 PNFs. **(A)** TEM image of RM-8 PNFs. Scale bar indicates 500 nm. **(B)** RM-8 PNFs are chemically stable for 10 days, as analyzed by HPLC. **(C)** ATR-FTIR spectrum of RM-8, showing spectral maxima in the amide I region indicating β-sheet structure. **(D)** RM-8 PNFs efficiently enhance transduction rates of GFP-expressing GALV-RV in Jurkat cells. Two days after transduction GFP+ cells were determined by flow cytometry. Shown are average values ( ± SD) of three independent experiments. **(E)** RM-8 PNFs efficiently enhance transduction rates of GFP-expressing RD114/TR-RV in HEK293T cells. Three days after transduction GFP+ cells were determined by flow cytometry. Shown are average values ( ± SD) of three independent experiments. **(F)** RM-8 PNFs efficiently enhance transduction rates of Luciferase-expressing VSV-G-LV in HEK293T cells. Two days after transduction infection rates were determined by measuring Luciferase signal. Shown are average values of triplicates (± SEM) of three independent experiments. **(G)** RM-8 PNFs efficiently enhance transduction rates of Luciferase-expressing RD114/TR-LV in HEK293T cells. Three days after transduction infection rates were determined by measuring Luciferase signal. Shown are average values of triplicates (± SEM) of three independent experiments. In D-F the numbers above the bars show the n-fold enhancement of infection relative to the average of controls without peptide. **(H)** Transduction protocol for using RetroNectin and RM-8 PNFs as transduction enhancer in an *ex vivo* gene transfer. Created with BioRender.com. **(I)** Viability of T cells was determined using trypan blue staining after 7 days. Shown are average values (± SD) of two donors. **(J)** RM-8 PNFs enhance retroviral transduction of T cells similar to Vectofusin-1 and RetroNectin. GALV-RV was incubated with indicated transduction enhancers before transducing activated T cells according to protocol of **(H)** GFP+ cells were determined by flow cytometry after 7 days. Shown are values of two donors. **(K)** RM-8 forms aggregates 1 day after transduction. Scale bar indicates 1000 µm and for the inset 200 µm. ATR-FTIR, Attenuated total reflection Fourier transform infrared spectroscopy; A.u., arbitrary units; centrif., centrifugation step; GALV, glycoprotein of gibbon ape leukemia virus; HPLC, high-performance liquid chromatography; LV, lentiviral vector; RD114/TR, chimeric envelope glycoprotein derived from feline leukemia virus with the cytoplasmic tail derived from the murine leukemia virus glycoprotein; RV; γ-retroviral vector; SEM, standard error of mean; TEM, Transmission electron microscopy; VSV-G, glycoprotein of vesicular stomatitis virus.

Due to its simplicity and cost-effectiveness in synthesis, we continued our further study with the shortest 8-mer peptide RM-8. PNFs formation of RM-8 was confirmed by transmission electron microscopy (TEM, **Figure 2A**). Furthermore, a high conversion rate (CR) of 97% was determined. The CR indicates the percentage of monomers that self-assemble into aggregated structures (40). To analyze the chemical stability of RM-8, respective PNFs were incubated for 10 min, 1 h, 24 h or 10 d at room temperature (RT) or 37°C, followed by high performance liquid chromatography (HPLC) analysis. Chromatograms show one peak at the same retention time for each time point (**Figure 2B**), indicating that RM-8 PNFs are chemically stable over time. Further biophysical analysis using attenuated total reflection fourier transform infrared (ATR-FTIR) spectroscopy revealed the amide I maxima at approximately 1632 and 1676 cm^-1^ (**Figure 2C**), which are characteristic for the presence of β-sheet structures (48).

Since all CAR-T cell therapies approved to date are manufactured using γ-retroviral and lentiviral vectors (13), we analyzed RM-8 PNFs’ ability to enhance these vectors in comparison to the commercially available transduction enhancer Vectofusin-1, which is a 26-mer amphipathic peptide (30). RM-8 PNFs increased transduction of GFP-expressing γ-retroviral vector pseudotyped with the glycoprotein of GALV, or from feline leukemia virus (RD114/TR-RV) in a similar range as Vectofusin-1, from around 4% to 60% GFP+ cells for GALV-RV (**Figures 2D**, **S5B**) and from around 2% to 25% GFP+ cells for RD114/TR-RV (**Figures 2E**, **S5C**) without showing cytotoxicity (**Figures S4B, C**). Of note, RM-8 PNFs promoted transduction of luciferase encoding LVs carrying the G protein of the Vesicular Stomatitis Virus (VSV-G) or RD114 more efficiently than Vectofusin-1 (**Figures 2F, G**).

To test RM-8 PNFs in a more clinically relevant setting, activated human CD4+ and CD8+ T cells were transduced with GALV-RV in presence of different transduction enhancers (RM-8, EF-C, Vectofusin-1 and RetroNectin), according to the protocol described in **Figure 2H**. After cultivation for seven days, viability and transduction efficiencies were determined. RM-8 PNFs allowed similar transduction rates of T cells of around 28% GFP+ cells as Vectofusin-1 and RetroNectin while not showing any cytotoxic effect (**Figures 2I, J**, **S5E**). However, during the T cell transduction process large µm-sized fibrillar aggregates were observed for RM-8 PNFs (**Figure 2K**).

### 3.3 Optimization of RM-8 to its derivate D4 by a structure-activity relationship study

**Figure 3:**
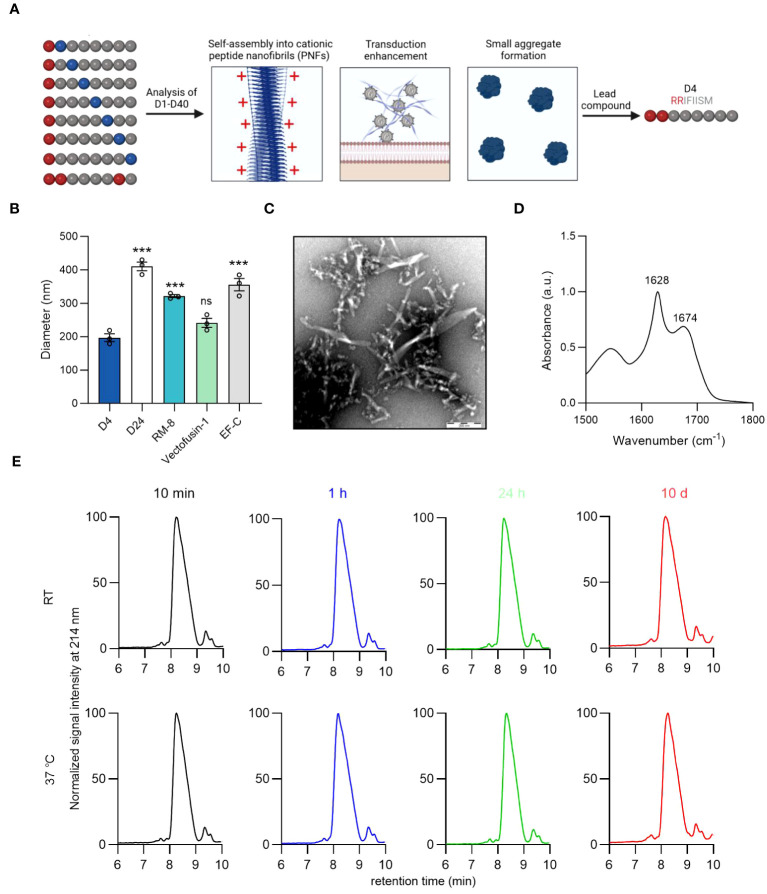
Identification of lead candidate D4 by a structure-activity relationship study of RM-8. **(A)** Optimization of RM-8 in regard to aggregate formation and transduction enhancing activity by a SAR study. 40 RM-8 derivatives (D1-D40) were generated by replacing the amino acids at position 2-8 with the following amino acids: isoleucine (I), proline (P), glycine (G), threonine (T), lysine (K) or arginine (R), which are shown in blue. Furthermore a derivative was generated containing three arginines (shown in red). The N-terminal R was not replaced as it is responsible for the positive charge of RM-8. Created with BioRender.com. **(B)** Diameter of D4, D24, RM-8, Vectofusin-1 and EF-C PNFs measured by NTA. The results shown are average values (± SD) of triplicates from three independent measurements. One-way ANOVA, Dunnett’s multiple comparison test. ***p ≤ 0.001 **(C)** TEM image of D4 PNFs. Scale bar indicates 200 nm. **(D)** FTIR spectrum of D4. The values in the plot refer to spectral maxima in the amide I region. **(E)** D4 PNF are chemically stable for 10 days. D4 PNFs (2 mg/ml) were incubated at room temperature and 37°C and after 10 min, 1 h, 24 h and 10 days, the chemical stability was analyzed using HPLC. Also, MALDI-TOF MS measurements were done on D4 PNFs after incubation for 10 days at RT and 37°C (**Figure S7**) to confirm that the monoisotopic mass did not change due to any modification. ATR-FTIR, Attenuated total reflection Fourier transform infrared spectroscopy; HPLC, high-performance liquid chromatography MALDI-TOF MS, Matrix assisted laser desorption ionization-time of flight mass spectrometry; NTA, nanoparticle tracking analysis; TEM, Transmission electron microscopy; SAR, structure-activity relationship.

The μm-scale RM-8 aggregates that formed in the T cell cultures could potentially hinder approval of RM-8 PNFs as a transduction enhancer in clinical settings. To address this, we fine-tuned RM-8 based on a structure-activity relationship (SAR) study, with a specific focus on minimizing aggregation (**Figure 3A**). For stability, we excluded amino acids prone to oxidation (phenylalanine, tryptophan, tyrosine, histidine, cysteine and methionine), intra- and inter-residue cyclization (asparagine, glutamine, aspartic and glutamic acid), and β-elimination (cysteine, serine and threonine) (49) from the SAR study, except for threonine, which was already present in RM-8 and did not induce instability.

We opted to evaluate a large peptide library composed of 40 RM-8 derivatives (**Table S2**). This was accomplished by mutating the amino acids at positions 2-8 of RM-8 with isoleucine (I), proline (P), glycine (G), threonine (T), lysine (K), and arginine (R). The C-terminal R remained unchanged due to its critical role in contributing to the positive surface charge and hence the enhancement activity. We picked the positively charged amino acids L and R with the prospect that an increase in net charge could diminish aggregation propensity through electrostatic repulsion (50), potentially leading to the formation of smaller, more beneficial aggregates. Our findings revealed that, with the exception of D17, D18, D25, and D26, all derivatives exhibited an amyloid-specific enhancement in ThT binding, as illustrated in **Figure S6A**. Additionally, out of the 40 RM-8 derivatives tested, only 10 displayed a positive ZP, as shown in **Figure S6B**. Most of the derivatives amplified γ-retroviral transduction rates in Jurkat cells comparably to RM-8 PNFs, without any noticeable cytotoxic effect (**Figures S6C, S6D**).

Microscopic imaging of derivatives with similar transduction enhancing activity to RM-8 showed no significant aggregation in D4 and D24 (**Figure S7**). Using nanoparticle tracking analysis (NTA), we found D4’s diameter to be around 200 nm, similar to Vectofusin-1, while D24, RM-8, and EF-C showed considerably larger diameters (**Figure 3B**). It is important to note that NTA can only measure particle sizes from about 30 to 1000 nm (51), thus making it unable to measure PNFs larger than 1 µm. This led us to identify D4 (RRIFIISM), where a threonine was replaced by an arginine at position 2, as the lead candidate from the SAR study. TEM analysis depicted that the D4 peptide assembled into short PNFs (**Figure 3C**). Additionally, a CR of 98% was determined, and ATR-FTIR spectroscopy analysis confirmed a high β-sheet content (**Figure 3D**).

Peptides can undergo modifications during storage that may affect their functionality (49). Given that D4 contains a serine residue prone to β-elimination (49), and the oxidation-sensitive amino acids methionine and phenylalanine (52, 53), we assessed its stability using HPLC and MALDI-TOF MS. HPLC analysis consistently showed a single peak with unchanged retention time across different time points (**Figure 3E**). Similarly, MALDI-TOF MS confirmed the consistent monoisotopic mass of D4 before and after a 10-day incubation (**Figure S8**).

### 3.4 D4 PNFs efficiently enhance γ-retroviral and lentiviral transduction of human T and NK cells

**Figure 4:**
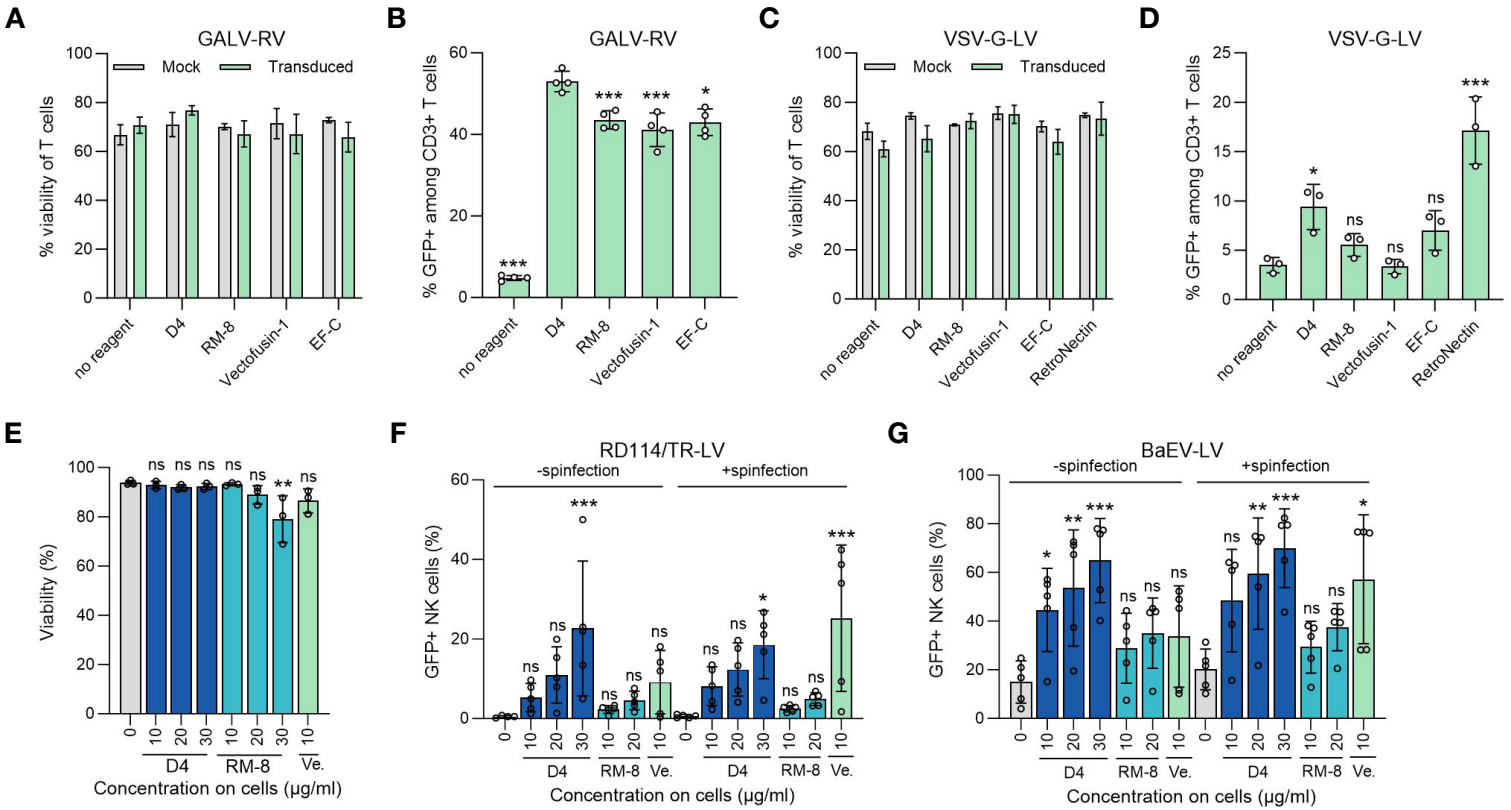
D4 as an efficient transduction enhancer for γ-retroviral and lentiviral transduction of human T and NK cells. **(A)** Viability of T cells was determined using trypan blue staining 7 days after GALV-RV transduction. Shown are average values (± SD) of four donors. Two-way ANOVA, Šidák’s multiple comparison test. No significant differences in viability between no reagent and the transduction enhancers were determined. **(B)** D4 PNFs more efficiently enhance GALV-RV transduction of T cells than RM-8, Vectofusin-1 and EF-C PNFs. GFP+ cells were determined by flow cytometry after 7 days. Data are presented as mean ± SD of four donors. One-way ANOVA, Dunnett’s multiple comparison test. **p ≤ 0.01, ***p ≤ 0.001. **(C)** Viability of T cells was determined using trypan blue staining 7 days after VSV-G-LV transduction. Shown are average values (± SD) of three donors. Two-way ANOVA, Šidák’s multiple comparison test. No significant differences in viability between no reagent and the transduction enhancers were determined. **(D)** D4 PNFs more efficiently enhance transduction rates of GFP-expressing VSV-G-LV in T cells than RM-8, Vectofusin-1 and EF-C PNFs. GFP+ cells were determined by flow cytometry after 7 days. Data are presented as mean ± SD of three donors. One-way ANOVA, Dunnett’s multiple comparison test. **p ≤ 0.01, ***p ≤ 0.001. **(E)** D4 PNFs do not reduce viability of human NK cells. Activated NK cells of three donors were incubated with different concentrations of D4 and RM-8 PNFs, and viability was determined by propidium iodide staining after 4 days. One-way ANOVA, Dunnett’s multiple comparison test. *p ≤ 0.05. Activated NK cells of five donors were transduced with a GFP-expressing LV pseudotyped with RD114/TR **(F)** or a baboon endogenous virus derived envelope (BaEV, **G**) with and without spinfection. GFP+ cells were determined by flow cytometry after 4 days. Shown are average values (± SD) of five donors. One-way ANOVA, Dunnett’s multiple comparison test. *p ≤ 0.05, **p ≤ 0.01, ***p ≤ 0.001. GALV, glycoprotein of gibbon ape leukemia virus; RD114/TR, chimeric envelope glycoprotein derived from feline leukemia virus with the cytoplasmic tail derived from the murine leukemia virus glycoprotein; LV, lentiviral vector; RV, γ-retroviral vector; Ve., Vectofusin-1; VSV-G, glycoprotein of vesicular stomatitis virus.

Our lead candidate, D4, underwent evaluation in a T cell transduction procedure. This process entailed incubating different transduction enhancers with a GFP-expressing GALV-RV, followed by transduction of activated T cells. After 7 days, we assessed both the viability and the percentage of GFP+ T cells. Importantly, neither D4 PNF nor the other transduction enhancers affected T cell viability (**Figure 4A**). Furthermore, D4 PNFs amplified γ-retroviral transduction from 5% to 53% GFP+ cells, a thus a slightly increased enhancing activity as compared to RM-8 PNF, Vectofusin-1, and EF-C PNFs (**Figure 4B**). As 4 of 6 authorized CAR-T cell products are generated using a lentiviral vector pseudotyped with the glycoprotein of vesicular stomatitis virus (VSV-G-LV) for CAR gene tranfer (13), we analyzed D4 PNF and other transduction enhancers in a T cell transduction process using a GFP-expressing VSV-G-LV. None of the transduction enhancer reduced viability of T cells and only D4 PNFs and RetroNectin significantly enhanced transduction rates (**Figures 4C, D**).

The limited efficiency of viral gene delivery in primary NK cells, which is less effective when compared to primary T cells, presents a noteworthy challenge in the application of NK-cell based immunotherapy (19). Therefore, we chose to test the potential of D4 PNFs as transduction enhancer for primary NK cells. To exclude cytotoxic effects of PNFs on NK cells, a viability assay using propidium iodide staining was performed. After a 4-day incubation period, the highest concentration of RM-8 (30 µg/ml) slightly reduced cell viability while D4 and Vectofusin-1 did not show any impact on cell viability (**Figure 4E**). Following this, activated NK cells were transduced with lentiviral vectors in the presence of different PNFs, both with and without spinfection. Transduction efficiencies were assessed after 4 days. Due to the inefficient enhancement of transduction rates in NK cells using VSV-G-LVs (54), RD114 was selected for pseudotyping. RD114 specifically binds to the sodium-dependent neutral amino acid transporter (ASCT2) (55, 56), which is expressed on hematopoietic cells (57). Without spinfection, D4 PNFs significantly increased transduction rates of RD114-LV from 0.4% to approximately 23% GFP+ NK cells, while RM-8 and Vectofusin-1 did not exhibit a significant increase at tested concentrations (**Figures 4F**, **S5G**). However, with spinfection, both D4 and Vectofusin-1 significantly enhanced lentiviral transduction rates (**Figures 4F**, **S5G**).

An alternative envelope, derived from the baboon endogenous virus glycoprotein (BaEV), which binds to ASCT1 and ASCT2 (44), was also considered. Without spinfection, all concentrations of D4 PNFs significantly increased transduction rates of BaEV-LV from 15% to approximately 65% GFP+ NK cells, while RM-8 and Vectofusin-1 were only slightly active (**Figures 4G**. **S5H**). The lentiviral transduction rates were additionally significantly enhanced by both D4 PNF and Vectofusin-1 when spinfection was employed (**Figures 4G**, **S5H**).

### 3.5 D4 PNFs elicit no inflammatory response

**Figure 5:**
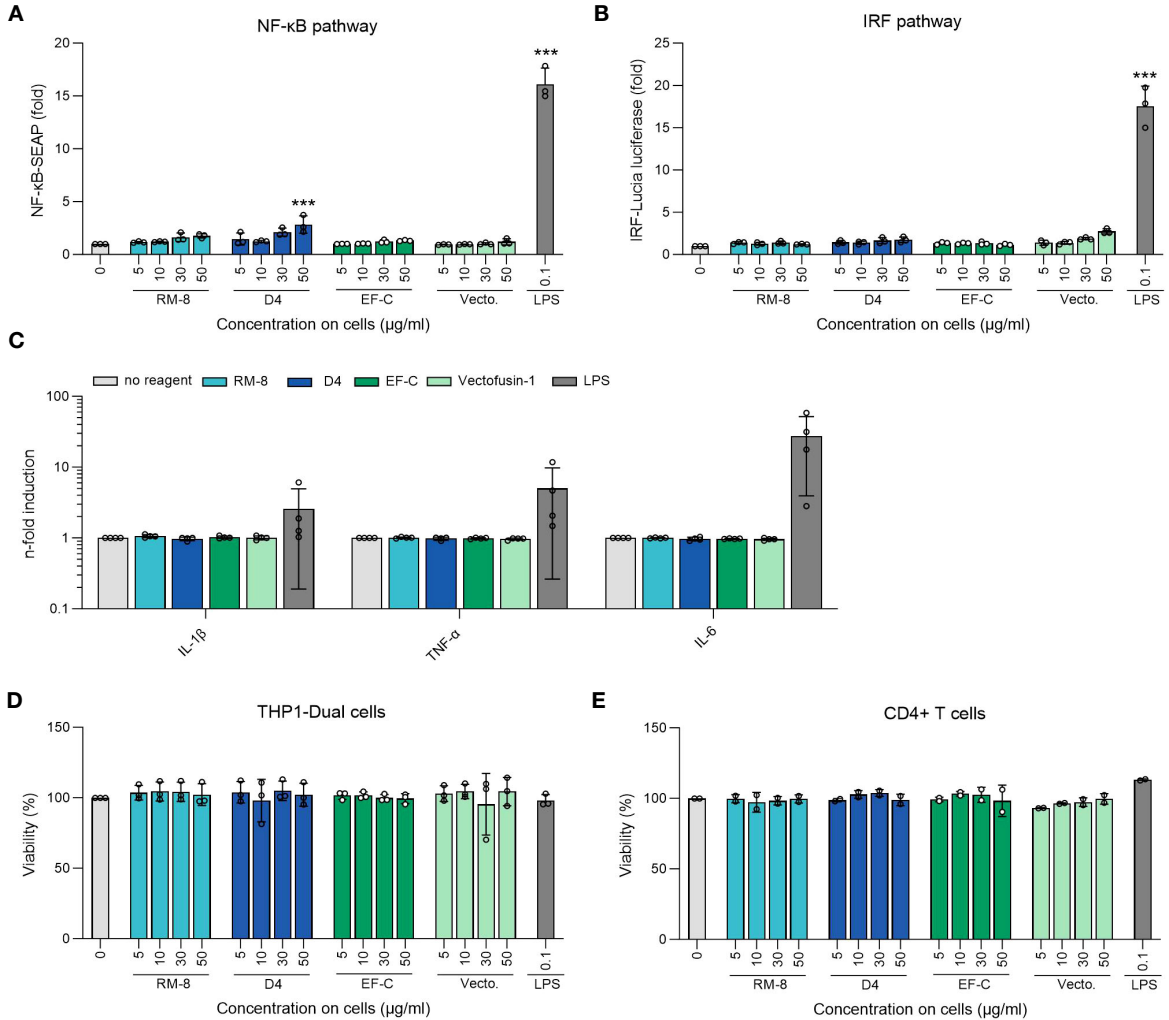
Analysis of inflammatory response of THP1-Dual and CD4+ T cells after PNFs stimulation. **(A)** Analysis of NF-κB pathway of THP1-Dual cells after PNFs and LPS stimulation. One-way ANOVA, Dunnett’s multiple comparison test. ***p ≤ 0.001. **(B)** Analysis of IRF pathway of THP1-Dual cells after PNFs and LPS stimulation. One-way ANOVA, Dunnett’s multiple comparison test. ***p ≤ 0.001. For A and B THP1-Dual cells, a monocyte-derived cell line, were treated with different PNFs (RM-8, D4, EF-C and Vectofusin-1) concentrations and 0.1 µg/ml LPS as a positive control. After one day the supernatants were simultaneously analyzed for activation of NF-κB pathway and the IRF pathway. Values were normalized to the PBS control. Shown are mean values (± SEM) of triplicate measurements from three independent experiments. **(C)** Analysis of different human inflammatory cytokines/chemokines after PNFs stimulation of CD4+ T cells using LEGENDplex Human Inflammation Panel 1. CD4+ T cells were treated with 30 µg/ml PNFs (RM-8, D4, EF-C and Vectofusin-1) and 0.1 µg/ml LPS as a positive control. After one day the cytokines/chemokines levels were analyzed using flow cytometry. Values were normalized to the untreated cells. Shown are mean values (± SEM) of duplicates of four donors. **(D)** Viability of THP1-Dual cells after PNFs and LPS stimulation. 24 h after stimulation viability was determined using CellTiter-Glo Luminescent Cell Viability Assay. Shown are mean values (± SEM) of triplicate measurements from three independent experiments. **(E)** Viability of CD4+ T cells after PNFs and LPS stimulation. 24 h after stimulation, viability was determined using CellTiter-Glo Luminescent Cell Viability Assay. Shown are mean values (± SEM) of duplicates of two donors. IL, interleukin; IRF, interferon regulatory factor; LPS, lipopolysaccharides; TNF-α, tumor necrosis factor alpha; SEAP, secreted alkaline phosphatase; Vecto., Vectofusin-1.

Aggregates in protein or peptide-based therapeutics often correlate with immunogenicity (58, 59). Given the potential application of D4 PNFs as a transduction enhancer in gene therapy, it’s crucial that they remain undetected by the innate immune system to prevent inflammation. To assess this, we examined the ability of D4, RM-8, EF-C, and Vectofusin-1 PNFs to trigger an immune response in THP1-Dual cells. This monocyte cell line is adept at simultaneously evaluating the NF-κB and interferon regulatory factor (IRF) pathways, both pivotal in the innate immune response (45). Different PNFs concentrations and lipopolysaccharides (LPS) as a positive control were incubated with THP-1 Dual cells for 24 h before supernatants were analyzed for activation of NF-κB and IRF pathway. Notably, only the 50 µg/ml concentration of D4 PNFs, which is not the concentration used in transduction experiments, showed significant activation of the NF-kB pathway (**Figure 5A**). As expected, LPS activated both pathways (**Figures 5A, B**). Next, we incubated PNFs with freshly isolated human CD4+ T cells for 24 h and subsequently used the LEGENDplex™ Human Inflammation Panel 1, a bead-based multiplex assay, to quantify human inflammatory cytokines/chemokines. Only LPS induced the expression of IL-1β, TNF-α and IL-6 of CD4+ T cells while the PNFs did not induce an inflammatory response (**Figures 5C**, **S9**). We also confirmed that neither the PNFs nor LPS exhibited cytotoxic effects (**Figures 5D, E**).

## 4 Discussion

The manufacturing and administration of CAR-T and CAR-NK cells involve labor-intensive procedures and significant costs, with the viral vector being a particularly expensive component (20). Enhancing target cell transduction efficiency can reduce the required quantity of viral vectors, potentially leading to cost savings (21); more importantly, it may enhance the success rate of the therapy by ensuring high transduction rates necessary for effective treatment (22). In this study, we demonstrate that D4 PNFs amplify retroviral transduction in primary T and NK cells, without inducing cytotoxicity or triggering inflammatory reactions. When juxtaposed with other transduction enhancers, D4 PNFs stand out for their simplicity and cost-efficiency. Their application is straightforward, involving a mere addition to the vector mix, sidestepping the labor-intensive coating or equipment-dependent spin infection processes associated with agents like RetroNectin or Vectofusin-1. Given its concise eight-residue structure, D4 can be synthesized more economically than lengthier peptides, such as Vectofusin-1. Moreover, D4 PNFs outperform Vectofusin-1 by bolstering VSV-G-LV mediated T cell transduction. These attributes of D4 PNF, including its prevention of extensive aggregate formation, non-toxicity, and non-inflammatory nature, position it as a pioneering tool for enhancing viral gene transfer. This potential is evident not just for research purposes but also holds promise for broader clinical applications.

Using an *in silico* screening approach, we searched for endogenous, non-immunogenic peptides that form into PNFs, effectively amplifying viral transduction rates. Leveraging amyloid prediction algorithms Tango, PASTA 2.0 and ZipperDB, a fibril-forming segment was determined within the IL-18 sequence. Based on this region, we synthesized five different cationic peptides named RS-12, RK-10, RM-8, RS-7 and FK-7 and subjected them to analysis. Among these, only RK-10 and RM-8 exhibited a positive ZP, making them effective in enhancing viral transduction. Notably, RM-8, an 8-mer peptide, swiftly self-organized into cationic, chemically stable β-sheet PNFs. Even though D4 contains a serine, which is sensitive to β-elimination (49), and the oxidation sensitive methionine and phenylalanine (52, 53), it stayed chemically stable over 10 days as shown by HPLC and MALDI-TOF analysis. It is crucial to mention that β-elimination of serine can impact peptide structure (60), and oxidation can occur due to photosensitization processes or the catalytic activity of transition metals (52, 61). Moreover, protein therapeutics’ oxidation, such as in methionine residues, can lead to adverse effects like diminished biological activity (62), reduced folding stability (61), and higher aggregation propensity (63). Therefore, RM-8 demonstrates chemical stability, a crucial factor for effective storage and development as a transduction enhancer for *ex vivo* gene transfer.

Notably, RM-8 PNFs demonstrated a superior ability to enhance VSV-G-LV transduction rates compared to Vectofusin-1. In fact, Vectofusin-1 showed no enhancement for VSV-G-LVs, aligning with prior research that suggested Vectofusin-1 doesn’t improve VSV-G-LV delivery into T and B cells (64, 65). This differential behavior can likely be traced back to the distinct entry mechanisms adopted by various vector systems. For instance, γ-retroviral vectors like GALV-RV employ a pH-independent approach for binding and fusion at the host cell membrane (66). In contrast, VSV-G-LV operates differently, undergoing endocytosis after binding to the host cell. This is followed by fusion with endosomal vesicles in acidic conditions, leading to the release of its payload into the cell’s cytoplasm (67). Therefore, it is suspected that Vectofusin-1 could potentially decrease the rate of endocytosis for vector particles that are attached to the cell surface (65). RM-8 PNFs achieved comparable γ-retroviral transduction rates of T cells as Vectofusin-1 and RetroNectin, without exhibiting any cytotoxic effects.

During the T-cell transduction procedure, we observed the emergence of large µm-sized fibrillar aggregates of RM8. Such aggregates could potentially induce unwanted effects if reintroduced into a patient during CAR T-cell therapy. Peptide aggregation is a common occurrence, culminating in either amorphous or amyloid-like fibrils (68, 69). This aggregation is dynamic, with amyloid-like fibrils capable of morphing into amorphous aggregates and *vice versa* (70). The exact influence of a peptide’s amino acid sequence on its propensity to form aggregates remains not fully elucidated (71). Yet, attributes like hydrophobicity, charge, β-sheet propensity, among others, are recognized to play a role in this process (68, 72–74). Our recent studies, employing a data-mining approach, revealed that high β-sheet content and hydrophobicity in amyloidal peptides are pivotal. They not only facilitate interactions with viral particles and cells but also promote the assembly of fibrils into larger µm-sized aggregates (47). In an effort to prevent the formation of large aggregates by RM-8, we developed the D4 peptide. D4 assembles into smaller fibrils, avoiding the formation of large aggregates during T-cell transduction. We speculate that D4’s heightened positive net charge, stemming from a threonine-to-arginine substitution, deters the creation of larger aggregates, possibly due to electrostatic repulsion (50). Interestingly, the chemically stable D4 PNFs displayed superior transduction rates in primary T and NK cells compared to both RM-8 and Vectofusin-1. We posit that the smaller aggregates, by offering a more expansive surface area, foster better interactions with viral particles and, subsequently, with cellular protrusions. This hypothesis aligns with our previous findings, where PNFs with numerous smaller µm-sized aggregates typically amplified transduction more effectively than their larger-aggregate-forming counterparts (47).

Immunogenicity refers to the ability of a molecule, typically foreign, to provoke either a humoral or cellular immune reaction from the patient (75). Considering the above, greater identity with endogenous molecules implies a lower risk of immunogenicity (76). An immune response against protein therapeutics often initiates with the activation of innate receptors, such as pattern recognition receptor (PRRs). This activation subsequently triggers the stimulation of antigen presenting cells (APC) (77–79). A notable link has been identified between aggregates in protein and peptide therapeutics and their immunogenic potential (58, 59). Additionally, chemical modifications, such as oxidation, can also trigger an immune response (80). When various PNFs were incubated with the THP1-Dual monocyte cell line, no activation of the NF-κB and IRF pathways was detected. In contrast, lipopolysaccharides (LPS), used as a positive control, activated both pathways through the toll-like receptor 4 (TLR4) (81). Moreover, the PNFs did not trigger any inflammatory response in CD4+ T cells, wheras consistent with literature, LPS stimulated the production of IL-1β, TNF-α and IL-6 (82). While our findings suggest that D4 PNFs are non-immunogenic, it’s imperative to conduct further studies to ascertain if they undergo cellular degradation during the CAR-T/NK cell process, ensuring no residual fibrils in the final therapeutic product.

In conclusion, D4 PNF stand out as an innovative and potent transduction enhancer, outperforming Vectofusin-1 in facilitating γ-retroviral gene delivery into T cells and lentiviral gene delivery into both T and NK cells. Its cost-effectiveness, attributed to its small size, combined with its user-friendly application that eliminates the need for additional centrifugation, positions it favorably for incorporation into automated GMP processes tailored for *ex vivo* viral transduction of primary cells. Consequently, D4 PNFs present a promising nanotechnological solution to augment viral transduction efficiencies in laboratory research and the production of CAR-T and CAR-NK cell therapies.

## Data availability statement

The original contributions presented in the study are included in the article/**Supplementary Material**. Further inquiries can be directed to the corresponding author.

## Ethics statement

The studies involving humans were approved by NK cells from healthy donors were isolated from buffy coats acquired from the blood bank of the University Hospital Leipzig; ethic vote number 327/22-ek. T cells from healthy donors were isolated from buffy coats acquired from the Blutspendezentrale Ulm; ethic vote number 151/22 FSt/bal. The studies were conducted in accordance with the local legislation and institutional requirements. The participants provided their written informed consent to participate in this study.

## Author contributions

LR-W: Conceptualization, Formal Analysis, Investigation, Project administration, Validation, Visualization, Writing – original draft. AR: Conceptualization, Formal Analysis, Investigation, Writing – review & editing. KK: Formal Analysis, Investigation, Writing – review & editing. TtW: Investigation, Writing – review & editing. LZ: Investigation, Writing – review & editing. AR: Formal Analysis, Investigation, Writing – review & editing. DS: Conceptualization, Writing – review & editing. SW: Supervision, Writing – review & editing, Funding acquisition. LS: Supervision, Writing – review & editing, Funding acquisition. TnW: Supervision, Writing – review & editing, Funding acquisition. DoS: Conceptualization, Funding acquisition, Supervision, Writing – review & editing. JM: Conceptualization, Funding acquisition, Project administration, Supervision, Visualization, Writing – original draft.
