# Supplementary material for: Optimized peptide nanofibrils as efficient transduction enhancers for in vitro and ex vivo gene transfer
Source: Front Immunol. 2023 Nov 9;14:1270243. doi: 10.3389/fimmu.2023.1270243 (PMC10666768; doi:10.3389/fimmu.2023.1270243)
Supplement: Supplementary file 1 [file DataSheet_1.pdf]

Supplementary Material

Optimized peptide nanofibrils as efficient transduction enhancers for *in vitro* and *ex vivo* gene transfer

Lena Rauch-Wirth[1], Alexander Renner[2], Kübra Kaygisiz[3], Laura Zimmermann[1], Tatjana Weil[1], Armando A. Rodríguez Alfonso[4,5], Desiree Schütz[1], Sebastian Wiese[5], Ludger Ständker[4], Tanja Weil[3], Dominik Schmiedel[2], Jan Münch[1, 4]*

[1] Institute of Molecular Virology, Ulm University Medical Center, Ulm, Germany

[2] Department for Cell and Gene Therapy Development, Fraunhofer Institute for Cell Therapy and Immunology (IZI), Leipzig

[3] Department Synthesis of Macromolecules, Max Planck Institute for Polymer Research, Mainz, Germany

[4] Core Facility Functional Peptidomics, Ulm University Medical Center, Ulm, Germany

[5] Core Unit of Mass Spectrometry and Proteomics, Ulm University Medical Center, Ulm, Germany

*** Correspondence:**
Jan Münch
jan.muench@uni-ulm.de

1 Supplementary Figures and Tables

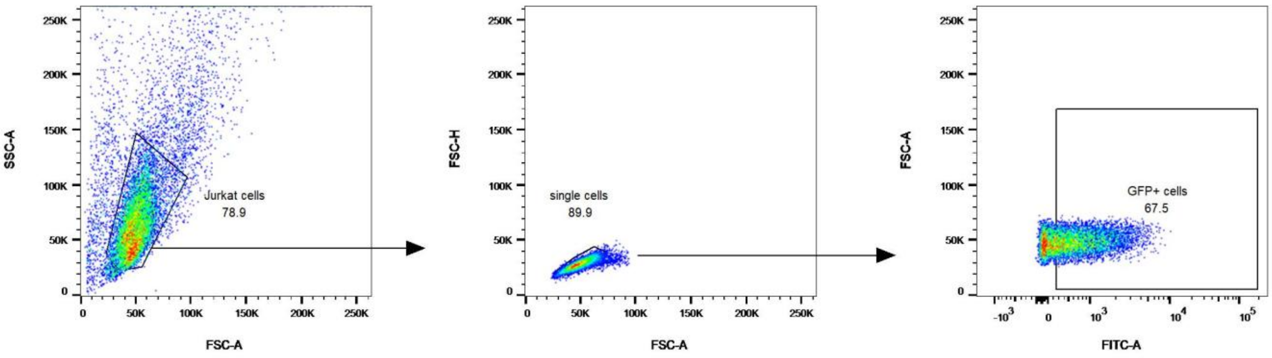

Figure S1. Gating strategy for analysis of GFP+ cells. Jurkat cells were initially gated on the basis of light scatter (SSC-A vs. FSC-A) and for singlets (FSC-H vs. FSC-A). GFP+ cells were measured using the FITC channel.

Table S1. Flow cytometry antibodies for the immunophenotype characterization of T cells.

Antibody	Fluorochrome	Company	Catalog No (Cat):
CD4	PerCP/Cyanine5.5	BioLegend	317428
Mouse IgG2b, κ Isotype Control	PerCP/Cyanine5.5	BioLegend	400337
CD8	PE	BD Bioscience	555367
Mouse IgG1, κ Isotype Control	PE	BD Bioscience	555749
CD11c	FITC	abcam	ab22540
Mouse IgG1 Isotype control	FITC	abcam	ab91356
CD3	BV421	BD Bioscience	562877
Mouse IgG1 k Isotype Control	BV421	BD Bioscience	562438

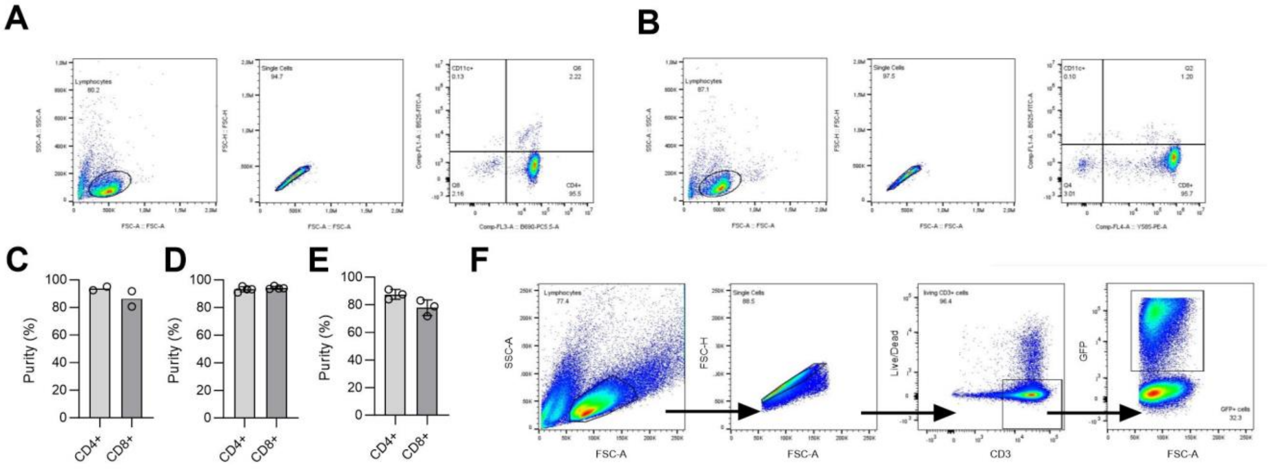

Figure S2. Gating strategy for purity and transduction efficiency of T cells. (A) Isolated CD4+ T cells were initially gated on the basis of light scatter (SSC-A vs. FSC-A) and for singlets (FSC-H vs. FSC-A). For purity CD4+ T cells (PC5.5) and CD11c+ cells (FITC) were measured. (B) Isolated CD8+ T cells were initially gated on the basis of light scatter (SSC-A vs. FSC-A) and for singlets (FSC-H vs. FSC-A). For purity CD8+ T cells (PE) and CD11c+ cells (FITC) were measured. (C) Purity of isolated CD4+ and CD8+ T cells of two donors used in T cell transduction experiment of Figure 2H, I. (D) Purity of isolated CD4+ and CD8+ T cells of four donors used in T cell transduction experiment of Figure 4A, B. (E) Purity of isolated CD4+ and CD8+ T cells of three donors used in T cell transduction experiment of Figure 4C, D. (F) Gating strategy for T cell transduction efficiency. T cells were initially gated on the basis of light scatter (SSC-A vs. FSC-A) and for singlets (FSC-H vs. FSC-A). Living CD3+ T cells were analyzed for GFP expression.

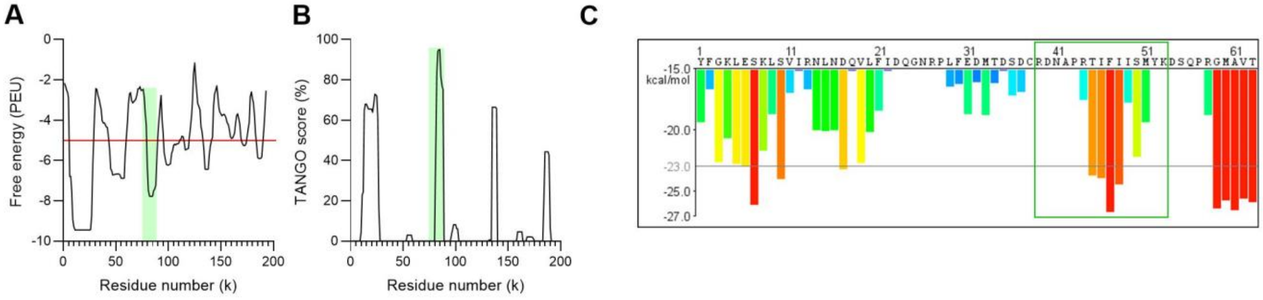

Figure S3. *In-silico* screening of human IL-18 by PASTA 2.0, Tango and ZipperDB. **(A)** Aggregation profile of IL-18 with an energy threshold of -5 PEU shown by the red line analyzed by PASTA 2.0. 1 PEU corresponds to 1.192 kcal/mol. Regions below the selected energy threshold are predicted to aggregate. **(B)** Aggregation propensity of IL-18 calculated with TANGO. A TANGO score (percentage β-aggregation per residue) of above 5 % in a window of at least 5 residues was chosen as a good predictor for aggregation. **(C)** Fibrillation propensity profile of IL-18 calculated by ZipperDB. The horizontal gray line shows the Rosetta energy of -23 kcal/mol. Hexapeptide with energies below this threshold shown as orange and red bars are predicted to have high propensity to form steric zippers. The calculation with PASTA 2.0 and TANGO is based on the IL-18 sequence (UniProtKB: Q14116) and the ZipperDB analysis was done with the IL-18 NMR structure (PDB: 1J0S). The predicted fibril-forming segment containing the five IL-18 peptides is marked in green at the position 75-89 (including propeptide) for A and B and at position 39-53 (without propeptide) for C. IL-18, interleukin-18; PEU, Pasta Energy Unit.

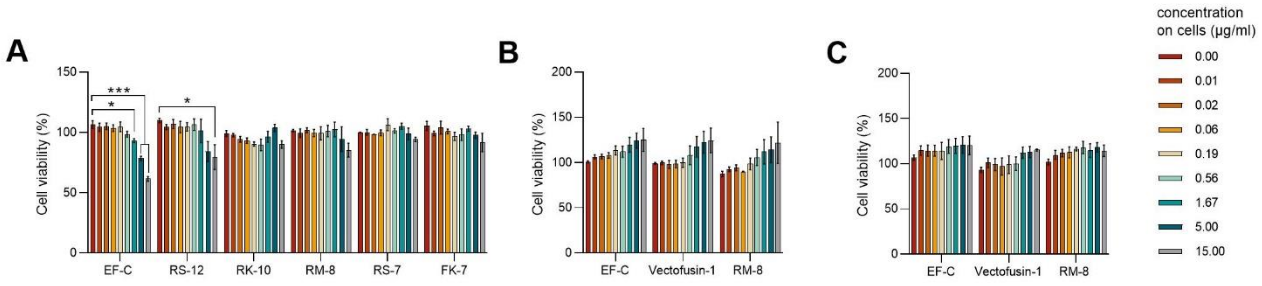

Figure S4. PNFs formed by IL-18 derived peptides are not cytotoxic**. (A)** TZM-bl cells were treated with different peptide concentrations. MTT assay was performed three days later. Shown are average values (± SEM) of triplicate measurements from three independent experiments. **(B)** MTT assay. **(C)** CellTiter-Glo® Luminescent Cell Viability Assay. Both assays were performed two days after the peptide treatment of Jurkat cells. Shown are average values (± SEM) of triplicate measurements from three independent experiments. The cell viability in % was calculated by normalizing sample values to untreated control which was set to 100 %. One-way ANOVA, Dunett's multiple comparison test Dunnett. **$p \leq 0.01$, ***$p \leq 0.001$. All concentrations without asteric are not significant.

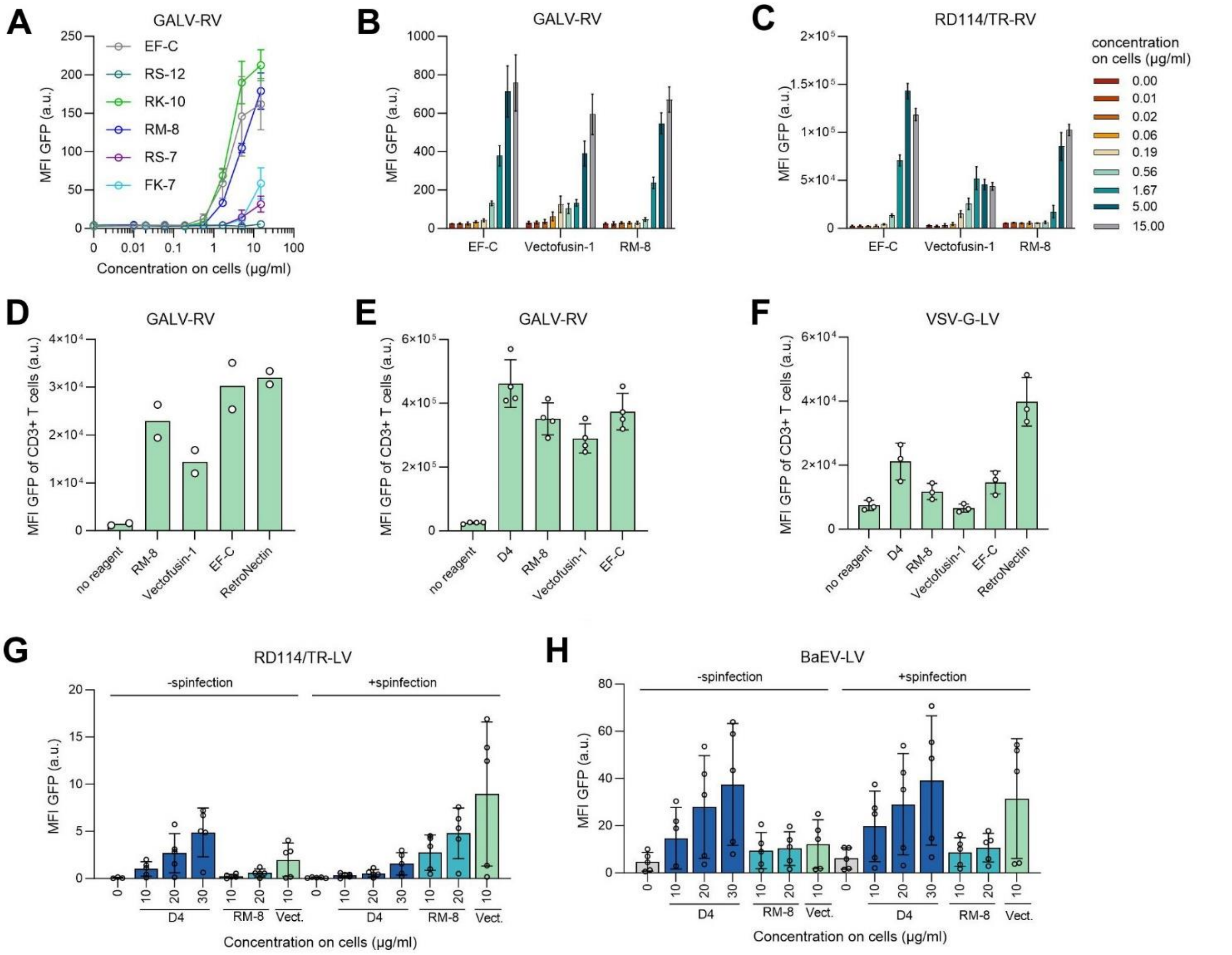

Figure S5. Corresponding MFI values to figures indicating the percentage of GFP+ cells in the main part of the paper. **(A)** RM-8 and RK-10 PNFs enhance GALV-RV infection of Jurkat cells (corresponds to Fig. 1F). **(B)** RM-8 PNFs efficiently enhance transduction rates of GALV-RV in Jurkat cells (corresponds to Fig. 2D). **(C)** RM-8 PNFs efficiently enhance transduction rates of GFP-expressing RD114/TR-RV in HEK293T cells (corresponds to Fig. 2E). **(D)** RM-8 PNFs enhance retroviral transduction of T cells similar to Vectofusin-1 and RetroNectin (corresponds to Fig. 2J). **(E)** D4 PNFs more efficiently enhance GALV-RV transduction of T cells than RM-8, Vectofusin-1 and EF-C PNFs (corresponds to Fig. 4B). **(F)** D4 PNFs more efficiently enhance transduction rates of GFP-expressing VSV-G-LV in T cells than RM-8, Vectofusin-1 and EF-C PNFs (corresponds to Fig. 4D). Activated NK cells of five donors were transduced with a GFP-expressing LV pseudotyped with RD114/TR **(G)** or a baboon endogenous virus derived envelope (BaEV, **H**) with and without spinfection (corresponds to Fig. 4F and 4G).

Table S2. Biochemical properties of RM-8 derivatives D1-D40. The table is displaying sequences, physical and chemical parameters of RM-8 and its derivatives. X^1 was replaced by Lysine (K). X^2 is selected from isoleucine (I), proline (P), glycine (G), threonine (T), lysine (K) or arginine (R). The amino acids replacing X^1 and X^2 are marked in bold red. pI, isoelectric point; MW, molecular weight; SEQ, sequence.

SEQ	Peptide		Length (aa)	MW (Da)	pI	Net Charge
-	RM-8	RTIFIISM	8	980.23	9.75	+1
**X**^{1}TIFIISM	1	**K**TIFIISM	8	952.22	8.75	+1
R**X**^{2}IFIISM	2	R**I**IFIISM	8	992.29	9.75	+1
	3	R**G**IFIISM	8	936.18	9.75	+1
	4	R**R**IFIISM	8	1035.32	12.00	+2
	5	R**K**IFIISM	8	1007.30	11.00	+2
	6	R**P**IFIISM	8	976.25	9.75	+1
RT**X**^{2}FIISM	7	RT**G**FIISM	8	924.13	9.75	+1
	8	RT**T**FIISM	8	968.18	9.75	+1
	9	RT**R**FIISM	8	1023.26	12.00	+2
	10	RT**K**FIISM	8	995.25	11.00	+2
	11	RT**P**FIISM	8	964.19	9.75	+1
RTI**X**^{2}IISM	12	RTI**I**IISM	8	946.22	9.75	+1
	13	RTI**G**IISM	8	890.11	9.75	+1
	14	RTI**T**IISM	8	934.16	9.75	+1
	15	RTI**R**IISM	8	989.24	12.00	+2
	16	RTI**K**IISM	8	961.23	11.00	+2
	17	RTI**P**IISM	8	930.17	9.75	+1
RTIF**X**^{2}ISM	18	RTIF**G**ISM	8	924.13	9.75	+1
	19	RTIF**T**ISM	8	968.18	9.75	+1
	20	RTIF**R**ISM	8	1023.26	12.00	+2
	21	RTIF**K**ISM	8	995.25	11.00	+2
	22	RTIF**P**ISM	8	964.19	9.75	+1
RTIFI**X**^{2}SM	23	RTIFI**G**SM	8	924.13	9.75	+1
	24	RTIFI**T**SM	8	968.18	9.75	+1
	25	RTIFI**R**SM	8	1023.26	12.00	+2
	26	RTIFI**K**SM	8	995.25	11.00	+2
	27	RTIFI**P**SM	8	964.19	9.75	+1
RTIFII**X**^{2}M	28	RTIFII**I**M	8	1006.31	9.75	+1
	29	RTIFII**G**M	8	950.21	9.75	+1
	30	RTIFII**T**M	8	994.26	9.75	+1
	31	RTIFII**R**M	8	1049.34	12.00	+2
	32	RTIFII**K**M	8	1021.33	11.00	+2
	33	RTIFII**P**M	8	990.27	9.75	+1
RTIFIIS**X**^{2}	34	RTIFIIS**I**	8	962.20	9.75	+1
	35	RTIFIIS**G**	8	906.09	9.75	+1
	36	RTIFIIS**T**	8	950.15	9.75	+1
	37	RTIFIIS**R**	8	1005.23	12.00	+2
	38	RTIFIIS**K**	8	977.21	11.00	+2
	39	RTIFIIS**P**	8	946.16	9.75	+1
R**X**^{2}IFII**X**^{2}M	40	R**R**IFII**R**M	8	1104.43	12.30	+3

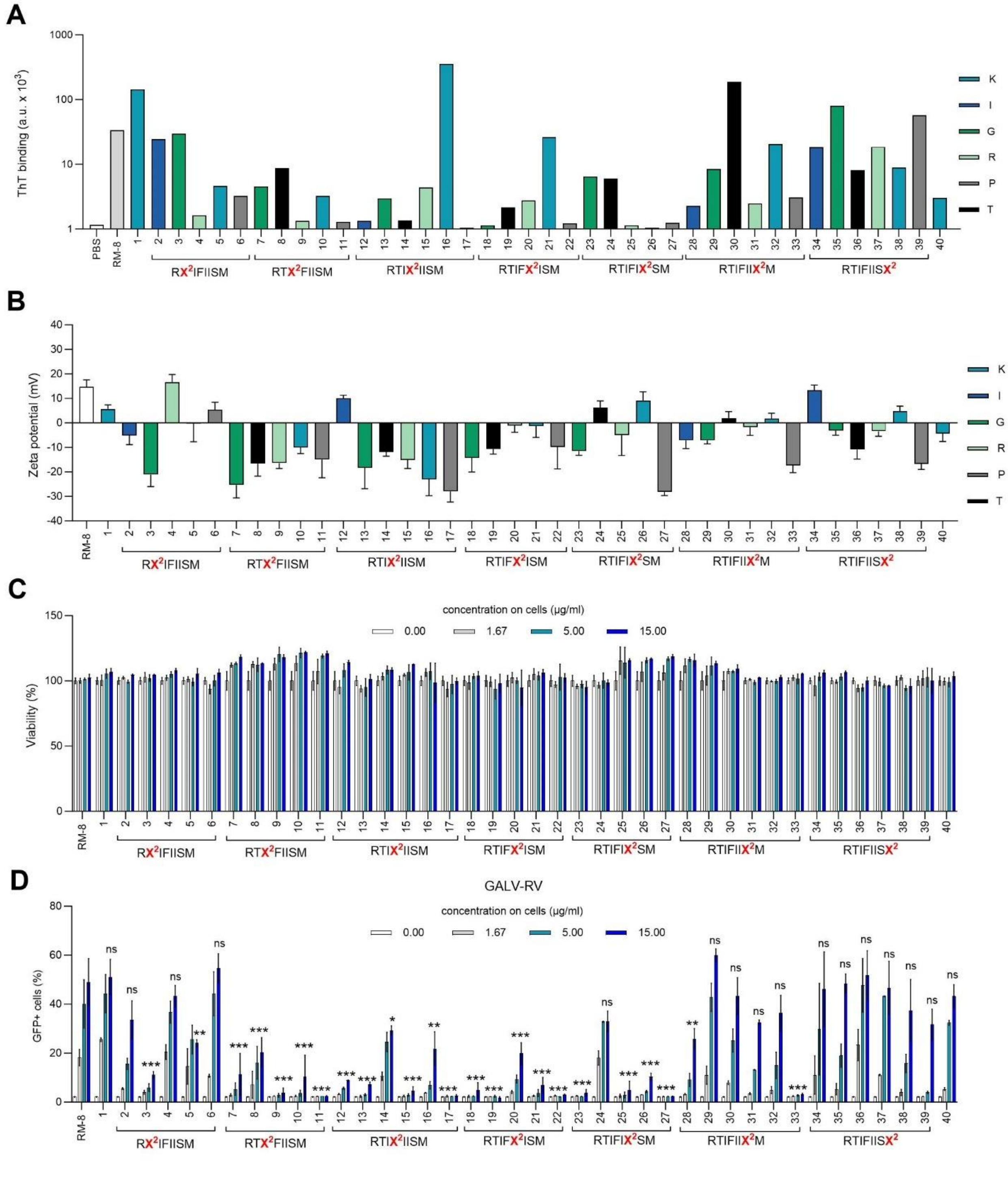

Figure S6. Characterization of RM-8 derivatives D1-D40. **(A)** ThT fluorescence of tested D1-D40 PNFs. Shown is one measurement from one experiment. **(B)** Zeta potential of D1-D40 PNFs. Shown are average values (± SD) of quintuplicate measurements. **(C)** CellTiter-Glo® Luminescent Cell Viability Assay. Both assays were performed three days after the peptide treatment of cells. The metabolic activity in % was calculated by dividing the averages of every peptide concentration by the values of cells in the absence of peptides. **(D)** GALV-RV was treated with PNFs and mixtures were used to infect Jurkat cells. GFP+ cells were determined three days later by flow cytometry. Shown are

average values (± SD) of triplicate measurements from two experiments. One-way ANOVA, Dunett's multiple comparison test Dunnett. *$p \leq 0.05$, **$p \leq 0.01$, ***$p \leq 0.001$. The mean of D1-D40 (15 µg/ml) was compared with the mean of RM-8 (15 µg/ml). X^2 of RM-8 was replaced with isoleucine (I), proline (P), glycine (G), threonine (T), lysine (K) or arginine (R). SD, standard deviation. GALV, glycoprotein of gibbon ape leukemia virus; RV; γ-retroviral vector; ThT, Thioflavin T.

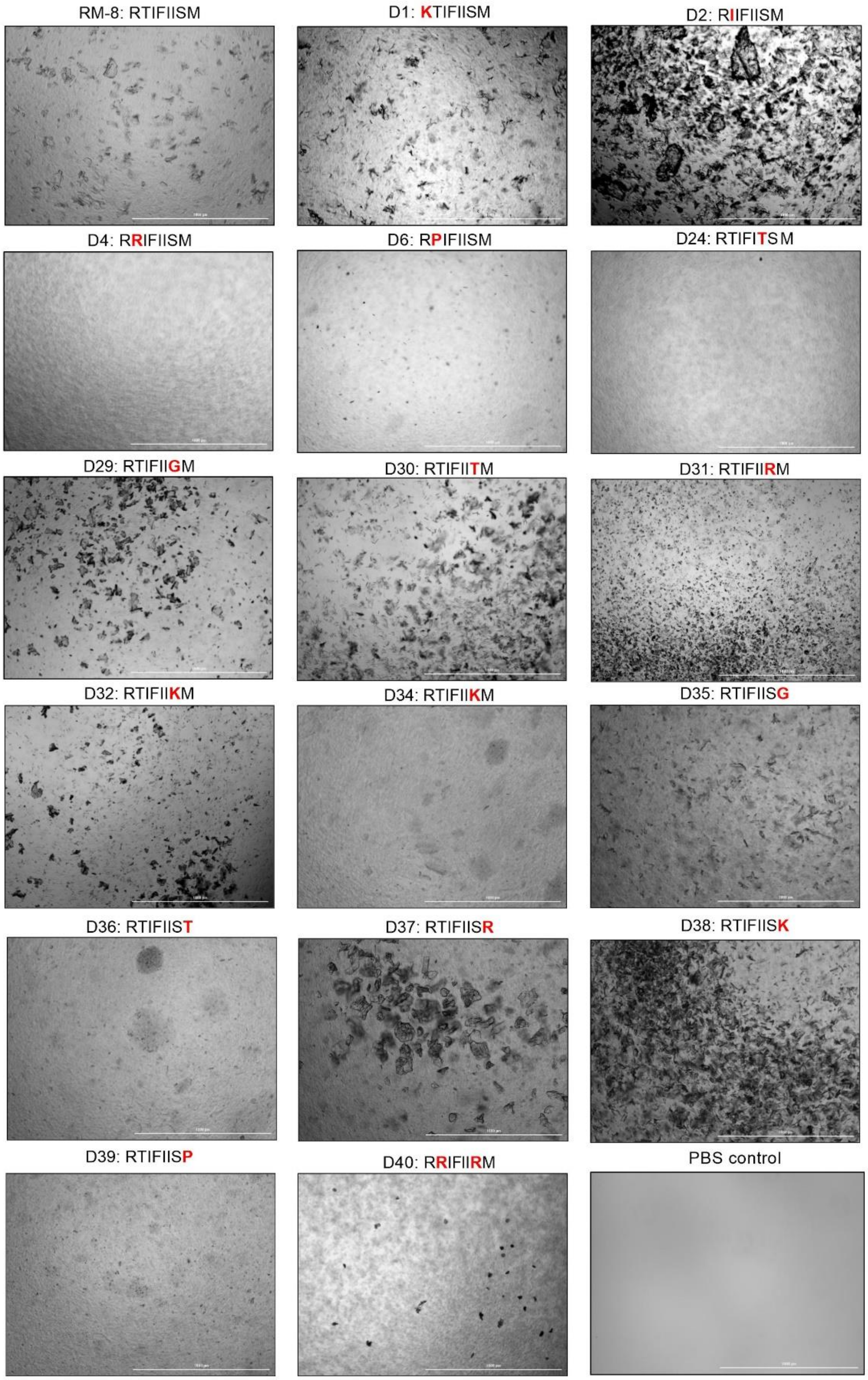

Figure S7. Microscopic images of RM-8 and its derivatives with a similar transduction enhancing activity. RM-8 derivatives (2mg/ml) were incubated for 10 min at room temperature (RT) before microscopic images were taken. The mutated amino acid is shown in bold red. Scale bar indicates 1000 µm.

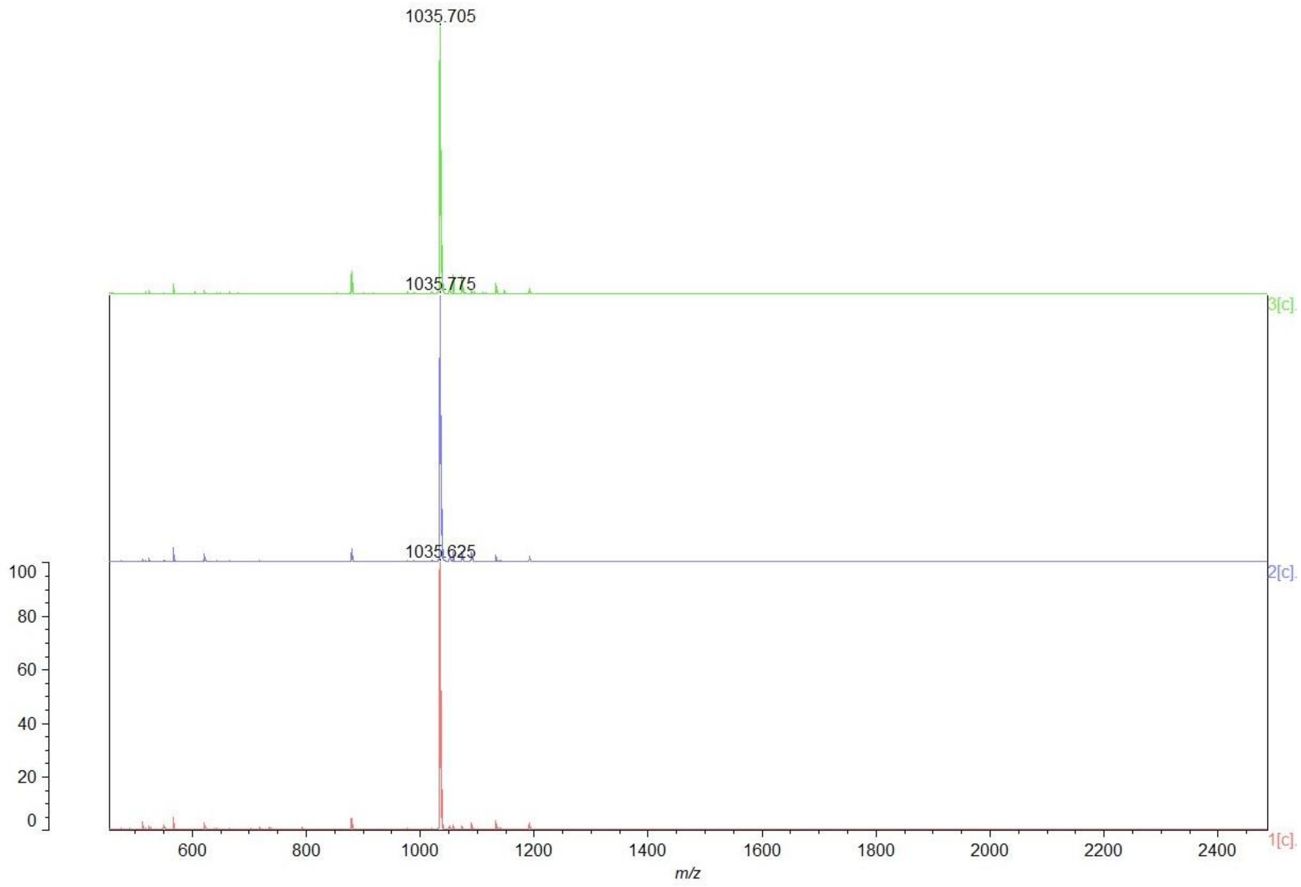

Figure S8. MALDI-TOF MS analysis of D4. Freshly solved D4 (green), D4 incubated at room temperature for 10 days (blue) and D4 incubated at 37 °C for 10 days (red) were analyzed. MALDI-TOF MS, matrix assisted laser desorption ionization-time of flight mass spectrometry.

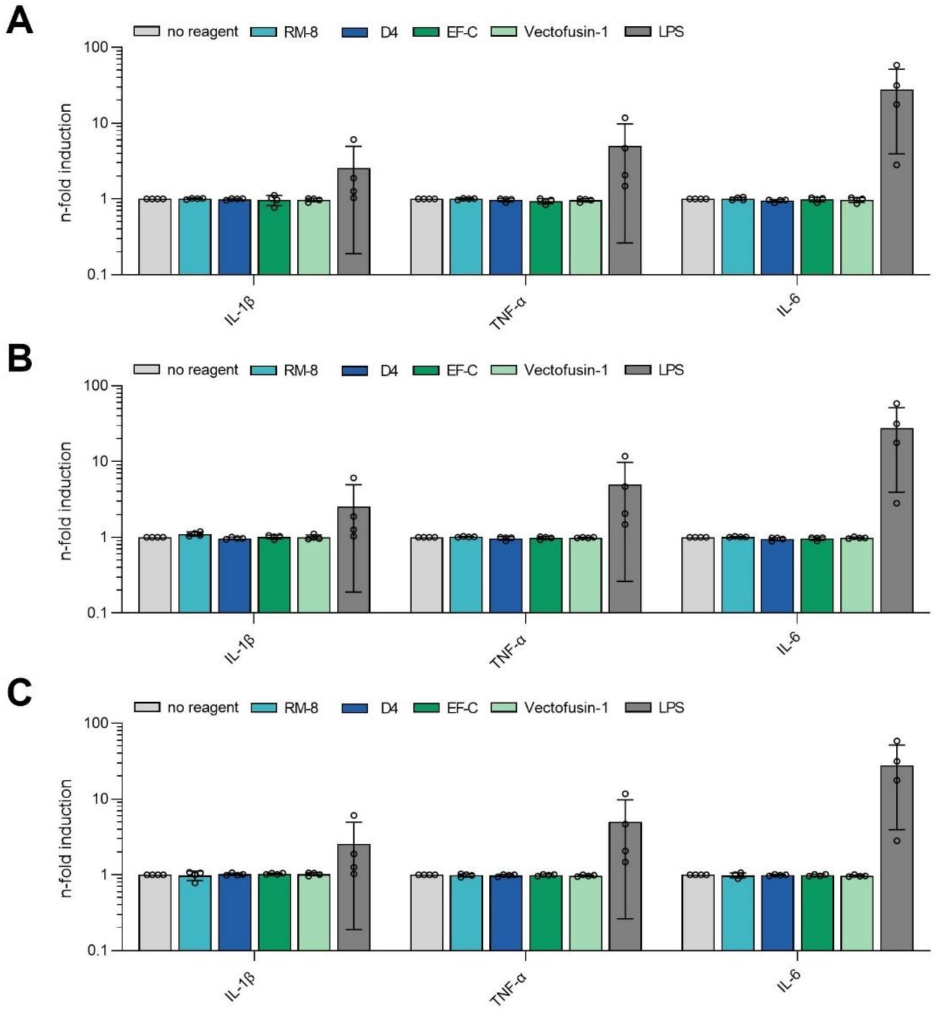

Figure S9. Analysis of inflammatory response of CD4+ T cells after PNFs stimulation. Analysis of different human inflammatory cytokines/chemokines after PNFs stimulation of CD4+ T cells using LEGENDplex Human Inflammation Panel 1. CD4+ T cells were treated with 5 µg/ml **(A)**, 10 µg/ml **(B)** and 50 µg/ml **(C)** PNFs (RM-8, D4, EF-C and Vectofusin-1) and 0.1 µg/ml LPS as a positive control. After one day the cytokines/chemokines levels were analyzed using flow cytometry. Values were normalized to the untreated cells. Shown are mean values (± SEM) of duplicates of four donors. IL, interleukin; LPS, lipopolysaccharides; TNF-α, tumor necrosis factor alpha.
